# Volcano–climate interactions: reframing the volcano–climate puzzle

**DOI:** 10.1007/s12210-026-01446-5

**Published:** 2026-08-13

**Authors:** Davide Zanchettin, Kameswar Rao Modali, Lauren Marshall, Shih-Wei Fang, Stergios Misios, Francesco S. R. Pausata, Angelo Rubino, Claudia Timmreck, Vito Zago

**Affiliations:** 1 Department of Environmental Sciences, Informatics and Statistics, University Ca’Foscari of Venice, Mestre, Italy; 2 Istituto Nazionale di Geofisica e Vulcanologia – Osservatorio Etneo, Catania, Italy; 3 Deutsches Klimarechenzentrum GmbH (DKRZ), Hamburg, Germany; 4 School of Earth and Environmental Sciences, University of St Andrews, St Andrews, UK; 5 College of Earth Sciences, Department of Atmospheric Sciences, National Central University, Taoyuan, Taiwan; 6 Research Centre for Atmospheric Physics and Climatology, Academy of Athens, Athens, Greece; 7 Department of Earth and Atmospheric Sciences, University of Quebec at Montreal, Montreal, QC Canada; 8 Max Planck Institute for Meteorology, Hamburg, Germany

**Keywords:** Volcanic eruptions, Volcanic stratospheric aerosol, Natural climate variability, Earth system models, Paleoclimate reconstructions

## Abstract

Explosive volcanic eruptions are major natural perturbations of the Earth system. By increasing stratospheric aerosol loading, they exert a dual radiative forcing: enhanced scattering of incoming solar radiation induces global surface cooling, while absorption of terrestrial radiation warms the lower stratosphere. Together, these radiative perturbations trigger complex dynamical responses within the coupled ocean–atmosphere–sea ice system, leading to diverse regional impacts. The resulting climate impacts are strongly event-specific, depending not only on the eruption’s magnitude, location, and timing, but also on the prevailing climate state and the phase of intrinsic variability. In this Perspective, we use the early nineteenth-century volcanic cluster—dominated by the unidentified 1809 eruption and the 1815 Mount Tambora eruption—as a natural test case to assess recent progress in volcano–climate research. We review and reassess advances in volcanic source characterization, aerosol forcing, climate response, and attribution, using new analyses of this volcanic cluster to illustrate how uncertainties propagate across these interconnected components. Taken together, recent advances indicate that volcanic climate impacts cannot be understood as deterministic responses to forcing alone, but emerge from the interplay between forcing characteristics, internal variability, and state-dependent feedbacks. We identify key remaining bottlenecks and outline future research directions, emphasizing the role of high-resolution modeling, data assimilation, AI-based methods and emulators, and probabilistic frameworks to drive further progress. Finally, we argue that addressing the volcano–climate puzzle will require coordinated engagement across the geoscience community to better constrain volcano–climate interactions, with the long-term goal of fully integrating volcanic forcing into Earth system science.

**Graphical abstract:**

**Figure:**
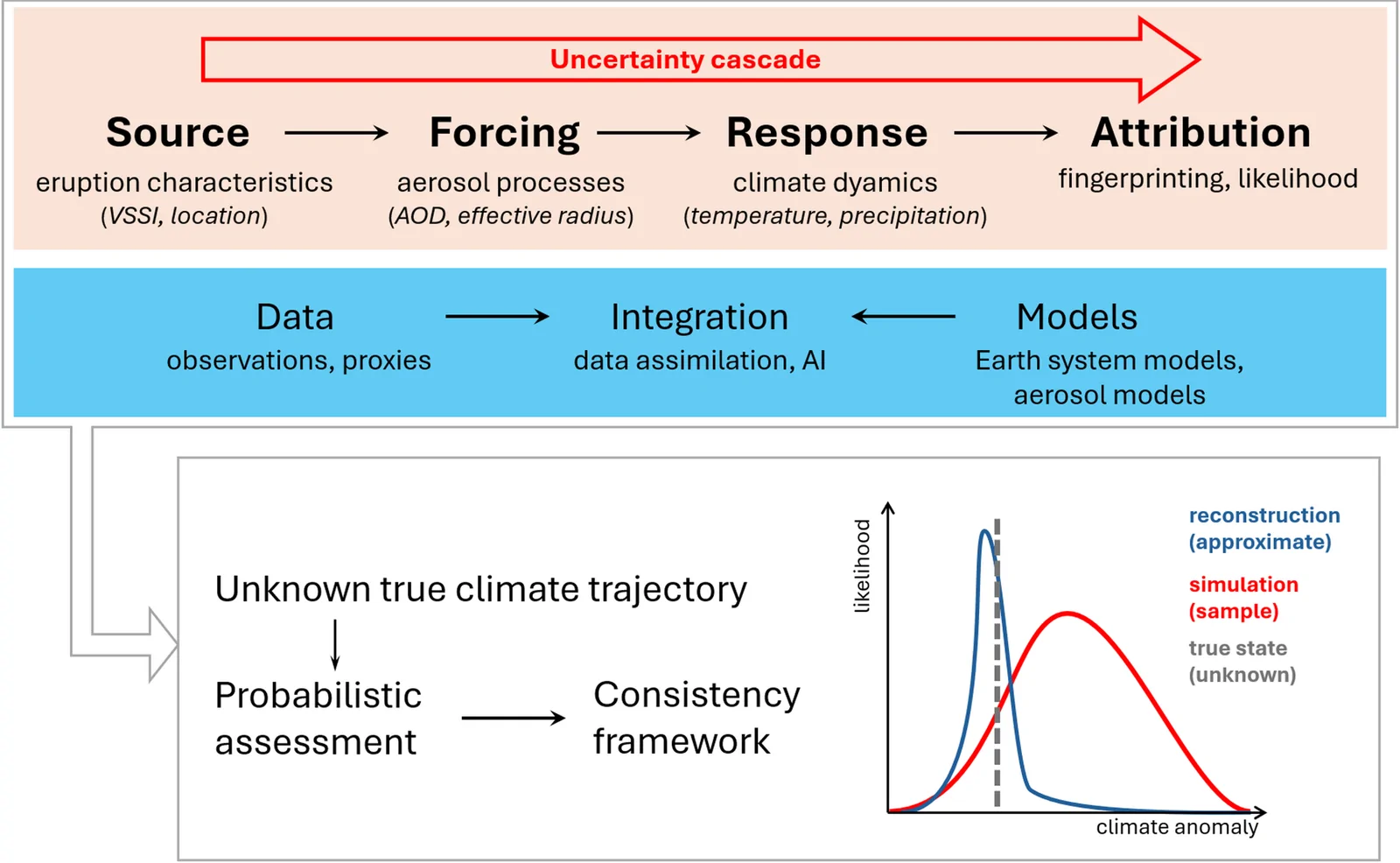

## Introduction

Explosive volcanic eruptions stand among the most formidable manifestations of nature, having shaped both our planet’s geological evolution and the course of human history. As a dominant natural climate forcing agent, explosive volcanism operates across a vast range of scales. While its immediate climatic impact is well known—driven by aerosol–radiation interactions, whereby volcanic aerosols primarily increase planetary albedo and induce surface cooling (e.g., Robock 2000)—the recognition that volcanism can profoundly alter climate across diverse timescales is a relatively recent paradigm shift, positioning it as a critical factor in contemporary climate discussions (e.g., Timmreck 2012; Zanchettin et al. 2016; Marshall et al. 2022). Yet, despite these advances, a central challenge remains: understanding how the climate system responds to impulsive volcanic perturbations.

The evolution of our understanding is reflected in the Intergovernmental Panel on Climate Change (IPCC) assessments. While the Fifth Assessment Report first introduced model-based evidence of prolonged volcanic influence (Myhre et al. 2013), the Sixth Assessment Report established with high confidence that explosive eruptions have been the primary driver of interannual-to-decadal climate variability over the past millennium (Eyring et al. 2021). This has placed volcano–climate interactions, i.e., the volcano–climate nexus, at the center of modern atmospheric and Earth system research (e.g., Chim et al. 2023; Aubry et al. 2026; D’Agostino et al. 2026), motivating what we refer to here as the volcano–climate puzzle: the challenge of deciphering how a nonlinear and state-dependent climate system responds to unpredictable, impulsive volcanic perturbations. In turn, this has fostered a dialogue on climate communication, highlighting the intrinsic and often unavoidable uncertainties that permeate volcanic activity and its global and regional impacts (Zanchettin 2023).

This perspective paper is rooted in the “Geosciences Nextgen: Earth Telescope” congress, held at the Accademia dei Lincei in Rome on 19–20 November 2025, which focused on exploring frontier discoveries and future challenges in the geosciences. Accordingly, this work highlights a decade of critical advancements in deciphering the volcano–climate puzzle. By focusing primarily on modeling approaches, we identify key research areas where significant progress is anticipated, providing a synthesis of the current state of the art and a roadmap for future investigations. We use the early nineteenth century—a period dominated by the close succession of two major eruptions: the unidentified 1809 eruption and the 1815 Mount Tambora eruption (e.g., Cole-Dai et al. 2009; Zanchettin et al. 2013; Brönnimann et al. 2019)—as a natural test case. By dissecting this interval, we aim to unravel the factors determining volcanically forced variability, delineate the hierarchy of propagated uncertainties, and ultimately provide constraints on the attribution of past climate variations and on uncertainties in future projections.

Given the scope of this Perspective, we briefly summarize the fundamental physical mechanisms linking volcanism and climate and refer the reader to comprehensive reviews covering the broader aspects of volcano–climate interactions (e.g., Robock 2000; Timmreck 2012; Kremser et al. 2016; Zanchettin 2017; Büntgen et al. 2020; Schmidt and Black 2022; Marshall et al. 2022; D’Agostino et al. 2026; and references therein).

In brief, from a climatic perspective, we are primarily concerned with explosive eruptions whose plumes penetrate the stratosphere. The critical parameter is the mass of injected sulfur dioxide (SO_2_), which undergoes chemical transformation to form sulfuric acid vapor. This vapor subsequently nucleates with water vapor to form new sulfate aerosol particles. The subsequent growth of these particles through condensation and coagulation determines their size distribution, ultimately controlling their optical properties and radiative efficiency. Large-scale stratospheric circulation then disperses the aerosol cloud: while tropical eruptions can achieve global coverage, extratropical emissions typically remain confined to the hemisphere of origin.

This volcanically enhanced stratospheric aerosol layer persists for 1–3 years following large tropical events before being removed via gravitational sedimentation and poleward transport. Volcanic forcing arises from aerosol–radiation interactions through two distinct mechanisms: first, the scattering of incoming shortwave radiation; and second, the absorption of longwave radiation. The latter reduces the Earth’s effective emissivity and induces a localized warming of the lower stratosphere. Consequently, the key metrics for characterizing volcanic aerosols are aerosol optical depth (AOD)—a measure of beam extinction by scattering and absorption—and the effective radius, which describes the aerosol size distribution.

These direct radiative effects trigger dynamical responses through a combination of bottom-up (surface cooling) and top-down (stratospheric heating) mechanisms. Within the coupled atmosphere–ocean–sea ice system, cascading feedbacks can prolong post-eruption climate anomalies well beyond the residence time of the aerosols themselves. Furthermore, for eruptions that do not penetrate the stratosphere or for the fraction of ash and precursors that remains confined to the lower atmosphere, indirect aerosol effects can play a crucial role. These tropospheric injections can significantly alter cloud microphysics—increasing cloud albedo and lifetime—and thereby introduce a further layer of complexity to the radiative forcing that is often decoupled from the stratospheric signal. Tropospheric aerosol effects are beyond the scope of this Perspective; interested readers are referred to specific papers on this topic (e.g. Malavelle et al. 2017; Wang et al. 2026; and references therein).

The core scientific challenge stems from the inherent rarity of strong volcanic eruptions; consequently, our direct observations are limited. Since the beginning of the satellite era, only a few major eruptions have significantly impacted the global climate, with the 1991 Mt. Pinatubo eruption remaining the preeminent observational benchmark and paradigm for interpreting volcanic impacts.

For eruptions predating the instrumental era, we must rely on indirect evidence and fragmented records. Assembling this volcano–climate puzzle requires four critical pieces: first, the volcanic source, i.e., the physical characterization of the eruption itself (e.g., magnitude, timing, emission height and geographical location); second, the forcing, i.e., the characterization of the volcanic aerosol cloud, including its evolution, transport, and optical properties; third, the climate response, i.e., understanding both the direct radiative effects of aerosols and the subsequent dynamical responses triggered within the coupled ocean–atmosphere–sea ice system; and fourth, attribution, i.e., disentangling volcanically forced mechanisms from internal climate variability and other external forcings, while accounting for the scarcity of direct meteorological observations and the lack of a well-constrained target.

Rather than representing independent scientific challenges, these elements form an interconnected cascade in which uncertainties propagate from volcanic source characterization through forcing generation and climate response to attribution. This uncertainty cascade, together with the principal constraints and remaining bottlenecks, is summarized in Table 1, which provides the conceptual framework for the remainder of this Perspective.

**Table 1:** Operational framework of the Volcano–Climate Puzzle

Puzzle Component	Dominant Uncertainty	Available Constraints	Remaining Bottlenecks	Global vs. Regional Impact
**Volcanic source**	Volcanic eruption magnitude; VSSI; plume injection height; eruption latitude and season.	Bipolar ice-core arrays (eVolv2k, IV2); historical/geological archives; petrological estimates.	Sulfate deposition to atmospheric burden conversion; limited temporal resolution in ice cores for closely spaced events (e.g., the 1809–1815 cluster); deposition rate uncertainties.	Direct control on hemispheric/global forcing magnitude; seasonality dictates the specific dynamical pathways to the extra-tropics (regional).
**Radiative forcing**	Aerosol microphysical processes (particle growth, coagulation, sedimentation); vertical structure of stratospheric heating vs. surface cooling.	EVA framework and eVolv2k, PMIP and VolMIP, interactive aerosol models (IsaMIP); modern instrumental analogues (e.g., 1991 Pinatubo).	Highly non-linear scaling in large-magnitude injections (e.g., radiative efficiency drop); forcing discrepancies between experiments/models with prognostics vs. prescribed aerosol.	Global-mean surface temperature is sensitive to net shortwave anomalies; longwave-driven stratospheric anomalies in meridional temperature gradient alter zonal winds (e.g., polar vortex, NAO), driving regional anomalies.
**Climate response**	Coupling between forced radiative anomalies and internal modes of climate variability (ENSO, NAO, AMOC); stratospheric-tropospheric coupling; initial state conditioning.	Single-model Large Ensembles; Multi-model intercomparisons (CMIP/PMIP); paleoclimate data.	Low signal-to-noise ratio at regional scales due to strong internal variability; model systematic features/biases in reproducing winter warming or zonal asymmetries; large uncertainties and structural discrepancies in paleoclimate datasets used as targets	Robust global thermal response; regional response (e.g., European anomalies) heavily masked by chaotic atmospheric circulation.
**Fingerprinting and attribution**	Quantifying the probabilistic contribution of the forced volcanic signal versus intrinsic, low-probability circulation anomalies (e.g., the 1816 European summer).	Proxy-based climate reconstructions; paleo-reanalyses, historical documentary evidence.	Model deficiencies in sampling the far tails of internal climate variability (*sampling density*); inherent uncertainties and structural diversity among proxy datasets.	Global attribution is robust (volcanic cooling); regional attribution requires a probabilistic framework to map extreme but physically plausible realizations.

The following sections examine each stage of this cascade along a narrative of escalating scientific complexity. After describing the data (Sect. 2), we progress from the volcanic source (Sect. 3) and the resulting radiative forcing (Sect. 4) to the climate response (Sect. 5), and finally to fingerprinting and attribution (Sect. 6). This sequence illustrates how the uncertainties accumulate across the four components of the volcano–climate puzzle and why the early nineteenth-century climate remains only partially constrained. Finally, we conclude in Sect. 7 with a forward-looking synthesis that identifies outstanding opportunities and future research trajectories. We argue that addressing the volcano–climate puzzle requires a shift in methodological, technological, and conceptual frameworks, supported by an integrated geoscience perspective.

## Data

To address the complexities of the volcano–climate puzzle, we utilize a hierarchy of ensemble simulations from various aerosol-climate and Earth system models, ranging from idealized to realistic conditions. This hierarchical framework allows us to disentangle the relative contributions of forcing uncertainty, model structure, and internal variability across a wide range of experimental configurations (Table 2). Specifically, we consider the following ensembles:

**Table 2:** Metadata and Operational Constraints of the Analyzed Super-Ensembles

Super-Ensemble	Model Components & Specifications	Number of models/model versions (M), realizations (*R*) and setups (S)	Volcanic forcing Implementation	Reference Baseline	Scientific Scope (S) and Experimental Constraints (C)
**VolMIP-Tambora **	Global aerosol microphysical models; multi-model; idealized Tambora simulation	M: 7; R: up to 5; S: 1 Models: UM-UKCA; CESM-WACCM; MAECHAM5-HAM point; MAECHAM5-HAM band; SOCOL-AER point; SOCOL-AER band; LMDZ-S3A band	Stratospheric sulfur injection, then interactive stratospheric aerosol calculations	Pre-industrial unperturbed climatology (SST, atmospheric greenhouse gas concentrations and tropospheric aerosol)	S: Estimate forcing (model diversity in aerosol processes) C: Source
**PMIP**	ESMs; multi-model; transient past1000/past2k simulations	M: 13 (7 PMIP3 and 6 PMIP4); R: 1 (3 for UKESM1); S: 4 (different volcanic forcing implementations of and forcing histories) Models: bcc-csm1-1; CCSM4; CSIRO-Mk3L-1-2; HadCM3; IPSL-CM5A-LR; MIROC-ESM; MPI-ESM-P; MPI-ESM1-2-LR; ACCESS-ESM1-5; INM-CM4-8; MIROC-ES2L; MRI-ESM2-0; UKESM1	Prescribed aerosol optical properties, except for UKESM1 and MRI-ESM2-0 (interactive stratospheric aerosol calculations). UKESM1 initializes the 1809 injection in October, while other models prescribe the eruption in January.	Transient full-forcing pre-industrial conditions	S: Response, Attribution C: Source (weak)
**volc-long-eq**	ESMs; multi-model; idealized Tambora simulation	M: 3; R: 9; S: 1 Models: MPI-ESM1-3-LR; MIROC-ES2L; CanESM5	eVolv2k, prescribed with EVA	Pre-industrial unperturbed climatology, supervised sampling of initial conditions	S: Response C: Forcing, initial conditions
**Tambora**	Single-model; volcanic-forcing-only simulations of 1809-Tambora eruptions	M: 1; R: 30; S: 3 (different stratospheric sulfur injection) Model: MPI-ESM1-2-LR	eVolv2k, prescribed with EVA	Transient conditions under volcanic forcing	S: Response C: Forcing (magnitude) ✖ *No Model Response diversity*
**E19C**	Single-model; transient past1000 simulations	M: 1; R: 20; S: 3 (different volcanic histories) Model: MPI-ESM1-2LR	no volcanic activity, eVolv2k and eVolv2k-D4, prescribed with EVA	Transient full-forcing pre-industrial conditions	S: Response, Attribution C: Source (volcanic history) ✖ *No Model Response diversity*
**EVA40**	Single-model; idealized Tambora-like simulation	M:1; R: 100; S: 3 (different eruption locations) Model: MPI-ESM1-2LR	EVA with 40 Tg S injection and controlled perturbations of spatial eruption parameters for tropical, Northern Hemisphere extratropical, and Southern Hemisphere extratropical	1991 conditions from transient full-forcing historical simulations	S: Response C: Forcing (spatial structure) ✖ *No Model Response diversity*

- The VolMIP-Tambora ensemble: a multi-model suite of idealized experiments where different global aerosol microphysical models follow a strict protocol to constrain sulfur injection (mass, height, and duration). This setup is designed to isolate model-specific differences in aerosol microphysics and transport (Zanchettin et al. 2016; Marshall et al. 2018; Clyne et al. 2021).
- The *volc-long-eq* ensemble, part of the VolMIP initiative (Zanchettin et al. 2016) contributing the sixth phase of the Coupled Model Intercomparison Project (CMIP6): this multi-model ensemble consists of volcanic-forcing-only simulations. It implements a rigorous protocol to constrain volcanic aerosol optical properties from a Tambora-like event using the Easy Volcanic Aerosol (EVA) module. EVA parameterizes the conversion from sulfur injection to AOD and effective radius through scaling relationships, enabling controlled comparisons across models while exploring forcing uncertainties (Toohey et al. 2016). This experiment uses the central sulfur injection estimate for the 1815 eruption from the eVolv2k reconstruction (Toohey and Sigl 2017), with nine-member ensembles available from MPI-ESM1-2-LR, MIROC-ES2L, and CanESM5.
- The Tambora super-ensemble: this single-model experiment (MPI-ESM1-2-LR) consists of three 30-member ensembles. It accounts for forcing uncertainty by testing three injection amounts (Best, Low, High) based on eVolv2k. Crucially, these simulations are initialized from a consistent set of conditions that already include the climate legacy and uncertainties of the 1809 eruption (Zanchettin et al. 2019; Schurer et al. 2019; Timmreck et al. 2021).
- The early-19th-century (E19C) super-ensemble: a comprehensive full-forcing suite based on MPI-ESM1-2-LR (Fang et al. 2022, 2023). It comprises three 20-member ensembles designed to evaluate different volcanic histories: no volcanic activity (NoVolc), the standard eVolv2k reconstruction, and the updated eVolv2k-D4 reconstruction, which includes the additional radiative contribution of small-to-moderate eruptions.
- The EVA40 super-ensemble: a single-model suite using MPI-ESM1-2-LR, representing a subset of a larger suite that explores climatic sensitivity to eruption latitude, magnitude, and location (e.g. Timmreck et al. 2024; Freychet et al. 2023; Meuer et al. 2024). It consists of three 100-member ensembles, each simulating a massive 40 Tg S injection (more than four times larger than the 1991 Pinatubo injection and compatible with the Tambora eruption within uncertainty). To isolate the role of spatial distribution, sulfur is injected at three different locations: tropical, Northern Hemisphere extratropical, and Southern Hemisphere extratropical (hereafter: TR, NH, SH). All simulations are initialized from 100 members of the MPI-ESM Grand Ensemble of historical simulations (Maher et al. 2019) in January 1991 leaving all forcing except the volcanic one unchanged. This provides a robust statistical framework for disentangling the forced signal from internal variability across different geographical forcing scenarios. A sibling set of experiments (eva0), identical to EVA40 but without volcanic forcing, is used as a baseline.
- The multi-model ensembles contributing to the *past1000* or *past2k* experiments in the third and fourth phases of the Paleoclimate Model Intercomparison Project (PMIP3 and PMIP4, Jungclaus et al. 2017) are full-forcing transient simulations using diverse volcanic forcing reconstructions. All models provide one realization, except UKESM1 for which three realizations are available and used here.
- Intrinsic variability is quantified through constant-forcing preindustrial simulations from CMIP6 *piControl* experiments.

Together, these ensembles provide a comprehensive experimental framework to investigate the propagation of uncertainty from volcanic source characterization to climate response.

## Volcanic source

Volcanic eruptions are episodic, stochastic, and inherently unpredictable; consequently, the resulting volcanic forcing is also highly uncertain and event-specific. The primary challenge in unraveling the volcano–climate nexus lies in constructing reliable volcanic histories that detail individual eruptions over time. This requires a precise characterization of source parameters, including timing, magnitude, geographical location, and duration, alongside plume properties such as total sulfur mass injection and peak injection height (Table 1). Uncertainty in these source parameters represents the first—and often dominant—source of uncertainty in the volcano–climate cascade.

Even for periods relatively close to the instrumental era, such as the early nineteenth century, reconstructing these histories necessitates the integration of disparate indirect evidence from multiple proxy sources. As a result, these reconstructions remain subject to continuous, and often substantial, refinements as new geological and ice-core data emerge.

For the 1815 Tambora eruption, timing and magnitude are well constrained through a combination of historical eyewitness accounts, geological evidence (including tephra deposits, caldera collapse structures, and stratigraphy), and geochemical reconstructions, establishing Tambora as a VEI-7 eruption and the largest volcanic event of the past 500 years (e.g., Oppenheimer 2003; Cole-Dai et al. 2009; Raible et al. 2016).

From a climatic standpoint, however, the key quantity is the volcanic stratospheric sulfur injection (VSSI). Reconstructing VSSI has therefore become a central objective of volcano–climate research. VSSI estimates for the 1815 Tambora eruption have evolved substantially as methods have improved, yet they remain uncertain due to limitations in both ice-core interpretation and scaling approaches. Early reconstructions relied on ice-core sulfate deposition from Greenland and Antarctica, assuming that polar sulfate fluxes could be scaled to global stratospheric aerosol burdens using relationships derived from well-observed eruptions such as Pinatubo (1991) or nuclear tracer experiments (Gao et al. 2007; Crowley & Unterman 2013). Subsequent advances in ice-core synchronization and reconstruction, such as those by Sigl et al. (2015), have significantly improved the temporal accuracy and global consistency of volcanic forcing estimates. More recently, the eVolv2k reconstruction (Toohey and Sigl 2017) has provided a major advance by combining synchronized, high-resolution bipolar ice-core records to derive time-resolved VSSI estimates, offering improved dating accuracy and a more physically consistent basis for reconstructing volcanic forcing. Nonetheless, multi-model analyses show that the relationship between deposited sulfate and atmospheric burden varies widely—by up to an order of magnitude across models—due to differences in aerosol microphysics, transport, and deposition processes that introduce substantial uncertainty into inferred sulfur emissions (Marshall et al. 2018).

For Tambora, recent sulfur injection estimates implemented in the eVolv2k and IV2 reconstructions converge around ~ 28 Tg S (~ 56 Tg SO_2_), reflecting a synthesis of bipolar ice-core records. However, plausible ranges remain broad (approximately 25–40 Tg S or 50–80 Tg SO_2_) because nonlinear processes—such as aerosol microphysics, enhanced sedimentation, and varying hemispheric transport—behave differently in large-magnitude injections compared to smaller instrumental-era eruptions like Pinatubo (Marshall et al. 2018, 2021).

In contrast to Tambora, the 1809 eruption remains a “climatic cold case”, as its existence and magnitude are inferred almost entirely from ice-core sulfate records, with no confirmed historical observations or identified source volcano (Timmreck et al. 2021). Bipolar sulfate signals in Greenland and Antarctic ice cores indicate a large tropical stratospheric injection, but key parameters—location, season, plume height, and aerosol distribution—remain uncertain. VSSI estimates vary depending on reconstruction methodology, with values of ~ 13–19 Tg S derived from ice cores and associated scaling approaches, placing the eruption between Pinatubo and Tambora in magnitude (Toohey and Sigl 2017; Timmreck et al. 2021). However, these estimates inherit uncertainties similar to those for Tambora: the conversion from ice-core sulfate deposition to atmospheric burden depends on assumptions about transport, hemispheric partitioning, and aerosol microphysics, which may vary substantially between eruptions. Additional uncertainty arises from the timing of the eruption, constrained only within several months based on high-resolution ice cores, and from the spatial structure of aerosol forcing, which strongly influences the climate response but cannot be uniquely reconstructed (Timmreck et al. 2021). More recently, explainable AI methods applied to post-eruption temperature patterns suggest a northern extratropical source for the unidentified 1809 eruption (Meuer et al. 2024). As such, the 1809 eruption represents an archetypal example of the volcano–climate puzzle, where key source characteristics remain fundamentally underconstrained.

In ice-sheet archives, identifying smaller eruptions remains challenging because sulfate deposition approaches background variability from non-volcanic sources (Toohey and Sigl 2017). Consequently, early reconstructions of nineteenth-century volcanism captured only the largest eruptions—notably the 1809 event and the 1815 Tambora eruption—while overlooking numerous smaller events. Recent high-resolution ice-core analyses now demonstrate that this period was characterized by a cluster of small-to-moderate eruptions, several of which can be linked to known VEI-4 events between 1812 and 1818 (Fang et al. 2023). These newly resolved events increase reconstructed stratospheric aerosol loading and reveal an emerging picture in which the early nineteenth-century volcanic forcing was more continuous and cumulative, rather than dominated solely by a few extreme eruptions.

This emerging picture challenges the traditional emphasis on isolated, extreme eruptions. Instead, it suggests that volcanic forcing may often be better understood as a temporally clustered and cumulative process, in which sequences of moderate eruptions can substantially modify the background radiative forcing and subsequent climate response.

## Radiative forcing

**Fig. 1:**
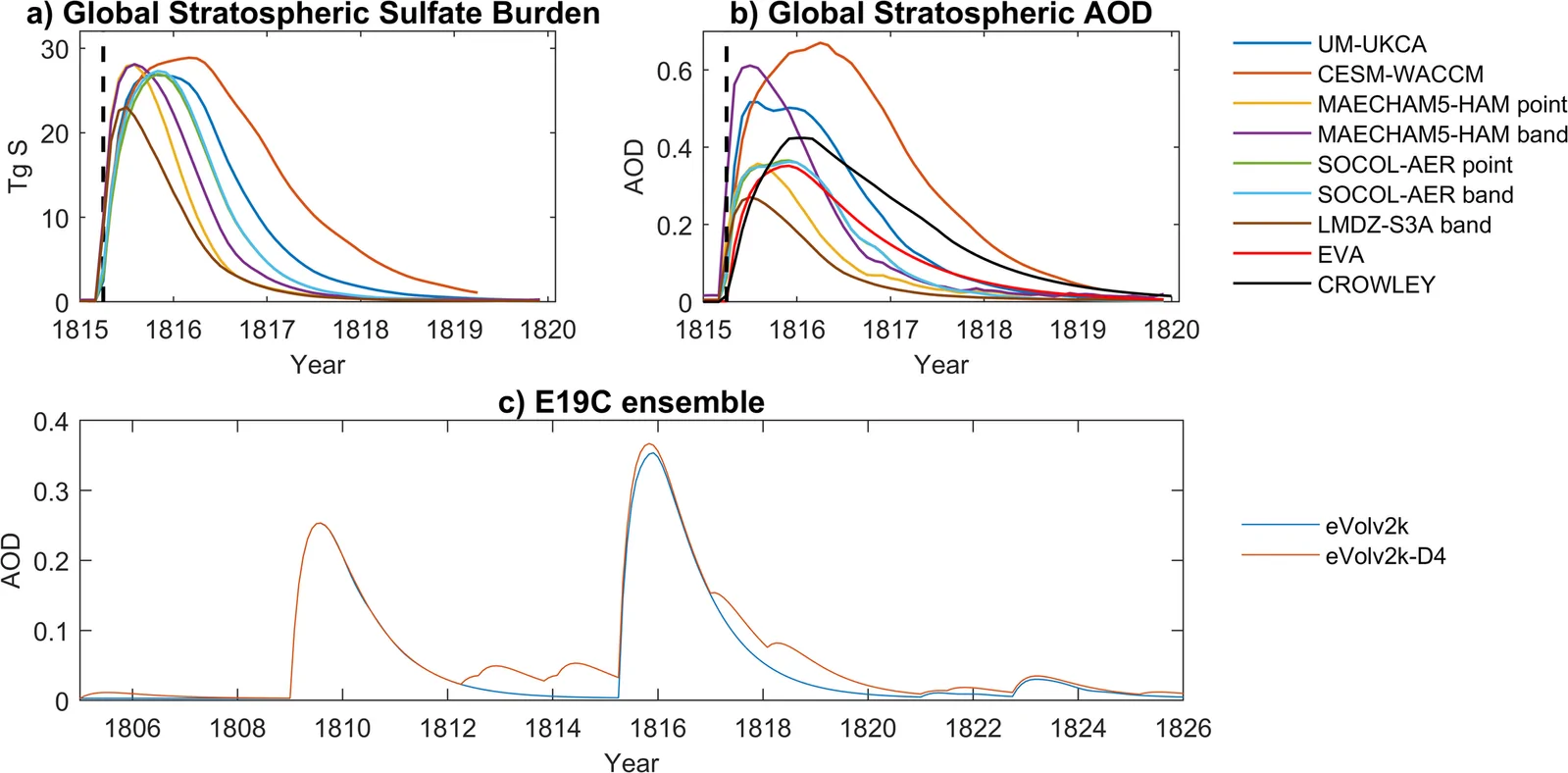
Comparison of volcanic aerosol parameters and historical reconstructions. (**a**) Global stratospheric sulfate burden and (**b**) aerosol optical depth (AOD) from the VolMIP-Tambora ensemble, simulating a 60 Tg SO_2_ injection. Panel b also includes the Crowley & Untermann (2013) reconstruction for comparison. (c) Evolution of early 19th-century global stratospheric AOD based on the eVolv2k and eVolv2k-D4 volcanic histories, as implemented in the E19C ensemble via the EVA forcing generator.

Radiative forcing represents a critical bottleneck in the volcano–climate puzzle, as uncertainties in sulfur injection propagate through aerosol microphysics to produce substantial differences in simulated aerosol optical properties and climate responses (Table 1). Even when models are forced with identical SO_2_ injections (e.g., 60 Tg for Tambora), they can produce substantially different stratospheric sulfur burdens and AOD due to differences in aerosol microphysics and chemistry. In the VolMIP-Tambora intercomparison, models show relatively consistent peak sulfate burdens (~ 27–29 Tg S, Fig. 1a), yet corresponding AOD values differ by up to a factor of two (Fig. 1b), demonstrating that AOD uncertainty is not controlled by sulfur mass alone (Clyne et al. 2021).

This divergence primarily reflects differences in effective particle size, which dictates radiative efficiency, as well as discrepancies in sulfate formation pathways and stratospheric transport. The MAECHAM5-HAM model provides a clear illustration of this sensitivity. Injecting SO_2_ at a single grid point, rather than distributing it across a latitudinal band, produces substantial differences in the simulated global stratospheric AOD (orange and purple lines in Fig. 1b). This highlights that increasing model complexity does not necessarily reduce uncertainty, as structural differences in aerosol representation can dominate the spread in simulated forcing.

Therefore, despite sacrificing process realism and being unable to fully capture feedbacks between aerosol microphysics, chemistry, and dynamics, simplified schemes such as EVA remain well suited for coordinated climate model experiments because they provide a consistent and computationally efficient representation of volcanic forcing, while avoiding the additional structural uncertainty associated with fully interactive aerosol–climate models. Accordingly, for the *past1000* and *past2k* experiments PMIP4 recommends the use of prescribed volcanic forcing datasets derived from ice-core sulfur reconstructions (e.g., eVolv2k) and translated into zonal and monthly mean aerosol optical properties using the EVA framework (Jungclaus et al. 2017).

However, the choice of the forcing dataset remains critical. The PMIP4 default forcing is largely based on eVolv2k reconstructions and, in practice, captures the dominant early nineteenth-century signal through the 1809 and 1815 eruptions. However, recent work (Fang et al. 2023) shows that incorporating the newly resolved events contributing to the early nineteenth-century cluster of small-to-moderate eruptions substantially modifies the temporal structure and persistence of the forcing (Fig. 1c), helping to explain prolonged cooling in the 1810–1820 decade. Despite these improvements, the eruption date for the 1809 eruption—as for all unidentified eruptions—is set to January 1st and its unknown location is assumed to be tropical. This highlights that while simplified schemes like EVA are effective tools, their fidelity ultimately depends on the completeness and accuracy of the underlying volcanic history.

**Fig. 2:**
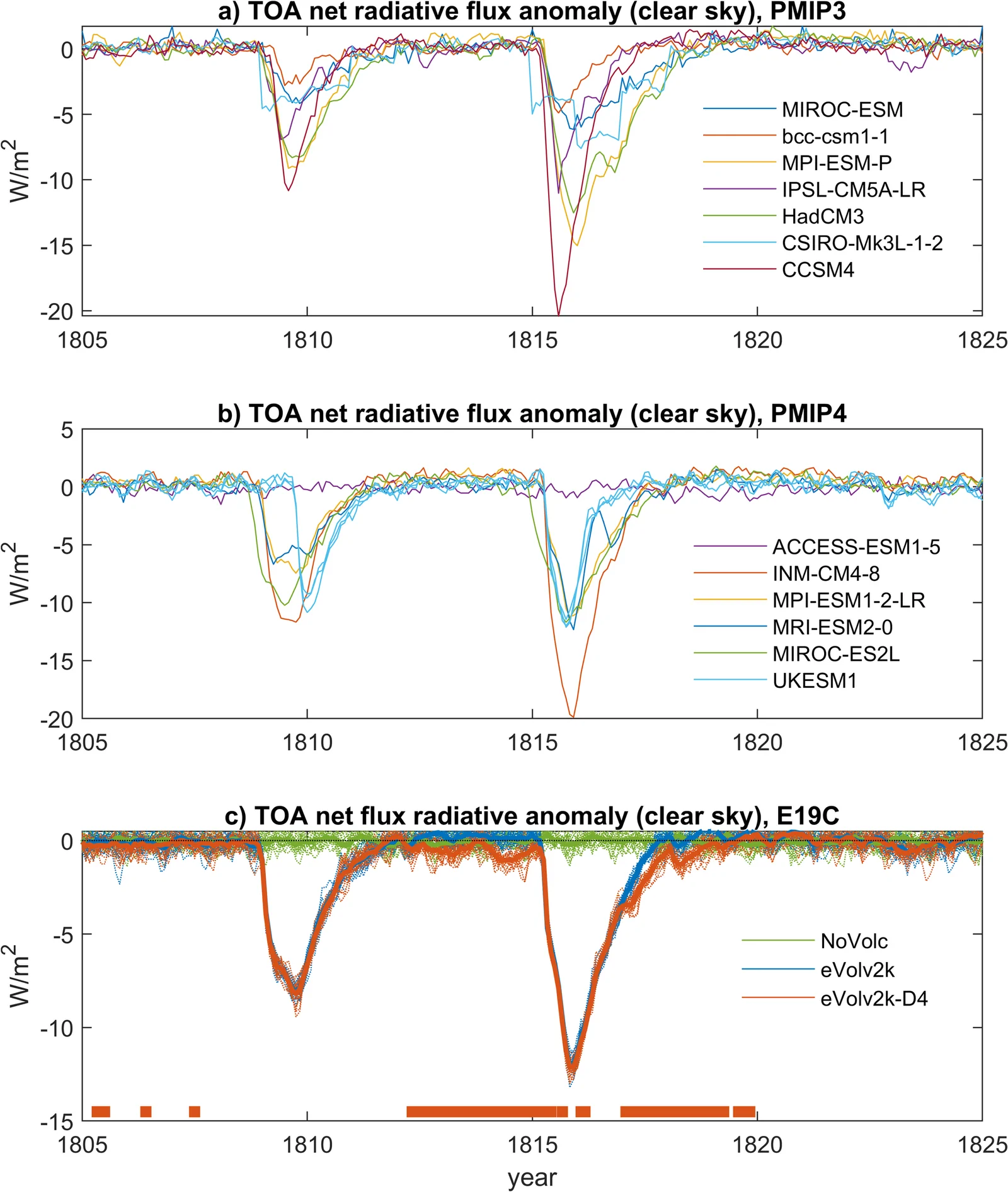
Simulated global-mean net top-of-atmosphere (TOA) clear-sky radiative fluxes. The panels show a hierarchy of experiments: (**a**) the PMIP3-*past1000* multi-model ensemble; (**b**) the PMIP4-*past1000/past2k* multi-model ensemble, with bluish colors indicating the two models with interactive aerosols; and (**c**) the E19C multi-forcing ensemble using MPI-ESM1-2-LR. Fluxes are calculated as the difference between incoming shortwave radiation (*rsdt*) and the sum of clear-sky outgoing shortwave (*rsutcs*) and longwave (*rlutcs*) radiation. For the E19C ensemble, dotted lines represent individual realizations, while thick continuous lines denote ensemble means. Brown squares at the bottom of panel (**c**) indicate months where the eVolv2k and eVolv2k-D4 ensembles differ significantly (*p* < 0.05) based on a Wilcoxon rank-sum test. Anomalies are calculated as monthly deviations from the 1780–1799 climatology for panels (**a**) and (**b**), and relative to the 1791–1810 period of the NoVolc control experiment for panel (**c**). In panel (**b**), ACCESS-ESM1-5 exhibits a negligible radiative response to the major eruptions, illustrating the substantial structural diversity that persists even within the PMIP4 ensemble. Panel (**a**) redrawn from Zanchettin et al. (2016)

To illustrate how forcing uncertainties propagate across the hierarchy of experimental designs introduced in Table 2; Fig. 2 compares simulated top-of-the-atmosphere (TOA) net radiative fluxes for the early nineteenth century, assuming clear-sky conditions. The comparison includes the PMIP3 multi-model ensemble, which uses early VSSI reconstructions (Gao et al., 2007; Crowley & Unterman, 2013), the PMIP4 *past1000* multi-model ensemble, which consistently uses the eVolv2k reconstruction, and the E19C ensemble, which is shown both including (eVolv2k-D4) and omitting (eVolv2k) small-to-moderate eruptions, highlighting their role in modulating the overall radiative balance alongside the dominant 1809 and 1815 signals.

The results clearly demonstrate that global radiative imbalances during volcanically perturbed periods remain substantially different across the considered hierarchy of experiments. In the PMIP3 multi-model ensemble (Fig. 2a), net TOA fluxes for the Tambora eruption exhibit a spread of a factor of four in peak values—a significant amplification compared to the twofold spread found in AOD. This inter-model divergence reflects propagating uncertainties rooted in the choice of VSSI reconstructions, associated AOD parameters, and model-specific forcing implementations. Furthermore, it incorporates the integrated effects of model differences regarding diverse atmospheric and surface processes, including direct radiative responses to volcanic aerosols and subsequent rapid adjustments. While these combined factors preclude any clear clustering of models based on the underlying volcanic reconstruction, caution is warranted: because this comparison spans different experimental designs and forcing reconstructions, it illustrates structural cross-ensemble diversity rather than a physical scaling between AOD and TOA radiative forcing.

The inflation of uncertainty from source to forcing is also evident in the smaller PMIP4 multi-model ensemble (Fig. 2b), despite the consistent use of the eVolv2k reconstruction across all models (Zhuo et al. 2022; Tao et al. 2025). This divergent behavior is apparent not only in the peak magnitude and timing of radiative anomalies for individual eruptions but also, crucially, in the timing and relative amplitudes produced by the 1809 and 1815 events. These inter-model differences are rooted in the forcing implementation: for instance, while MIROC-ES2L and MPI-ESM1-2-LR rely on prescribed optical properties from the EVA generator, MRI-ESM2-0 and UKESM1 prognosticate stratospheric aerosol evolution directly from precursor sulfur emissions.

The model-specific aerosol behavior in UKESM1—particularly the distinct spatial distribution of its volcanic cloud—explains why its post-Tambora radiative imbalance recovers more rapidly than in other ensemble members. Furthermore, the implementation of the 1809 unidentified eruption in UKESM1 as an October event (rather than an assumed January event, as in other models) highlights how choices in event dating are critical. Such a shift in injection timing can significantly alter the seasonal evolution and transport of the volcanic aerosol cloud, thereby modifying the cumulative radiative forcing and the potential for nonlinear interference within the early-nineteenth-century volcanic cluster (Marshall et al. 2025). Overall, these results demonstrate that uncertainties in volcanic forcing arise not only from source reconstruction, but also from model-dependent implementations of aerosol properties.

Refining volcanic histories to include small-to-moderate eruptions can significantly alter the simulated planetary radiative response within the same model. This is illustrated in Fig. 2c: in the E19C super-ensemble, the eVolv2k-D4 forcing exhibits markedly stronger TOA clear-sky net energy losses compared to the eVolv2k forcing, directly coinciding with the inclusion of additional eruptions in the 20-member MPI-ESM1-2-LR ensembles. Notably, while these radiative imbalances rapidly reconverge once the aerosol loading from the additional eruptions dissipates, this does not imply that the surface climatic signature is equally short-lived. As discussed in the following sections, coupled ocean–atmosphere–sea ice dynamics and the ocean’s thermal inertia can translate these transient radiative perturbations into more persistent climate signals.

Ultimately, the diversity of radiative forcing responses across models and experimental designs demonstrates that uncertainty at the forcing stage arises from multiple interacting processes rather than a single source. These uncertainties propagate nonlinearly into the subsequent climate response, setting the stage for the wide range of outcomes explored in the following section.

## Climate response

### From forcing to climate response

**Fig. 3:**
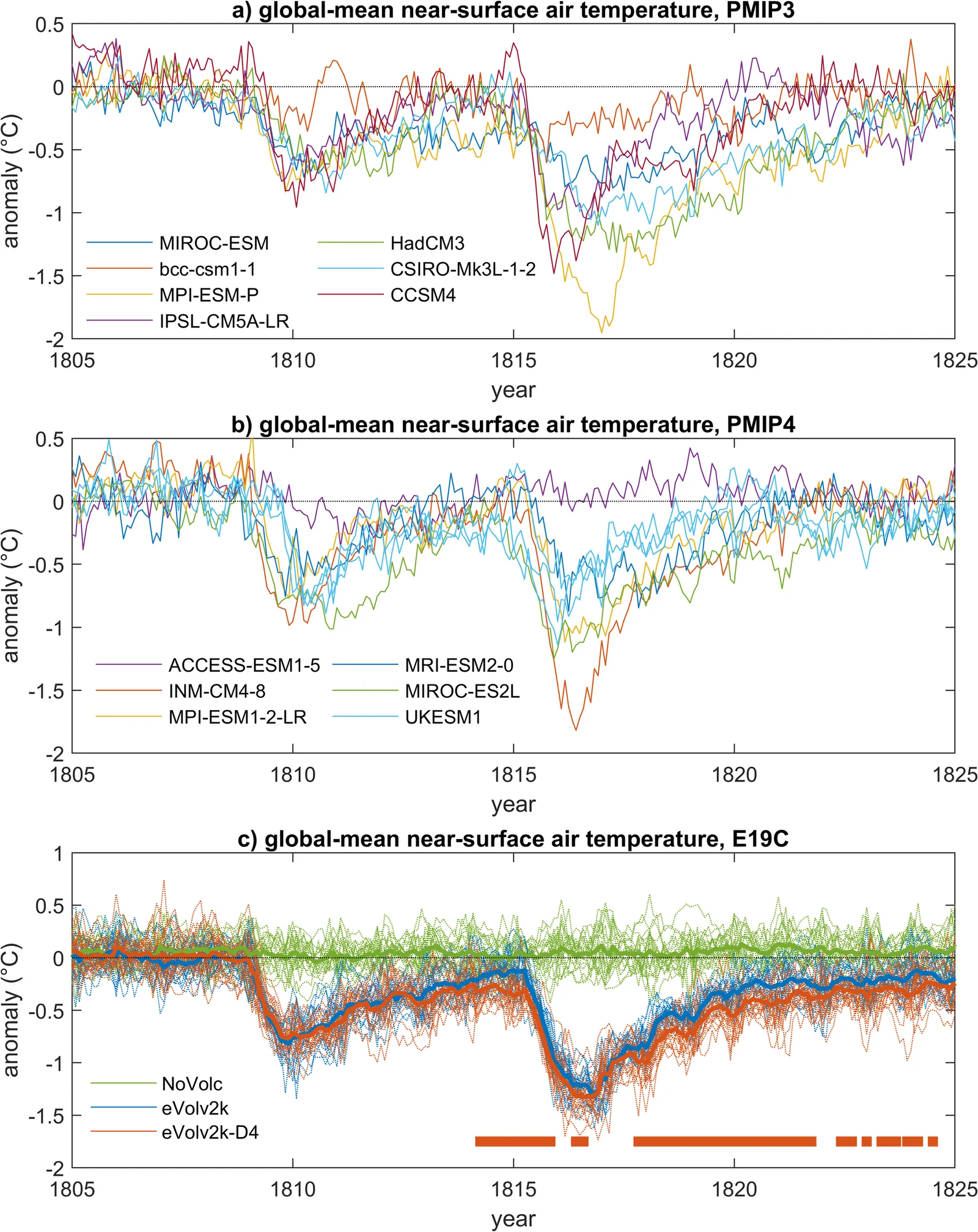
Simulated global-mean near-surface air temperature response. Time series of 2-meter (1.5-meter for UKESM1) air temperature (*tas*) across a hierarchy of experiments: (**a**) the PMIP3-*past1000* multi-model ensemble; (**b**) the PMIP4-*past1000/past2k* multi-model ensemble, with bluish colors indicating the two models with interactive aerosols; and (**c**) the E19C single-model (MPI-ESM1-2-LR) multi-forcing ensembles. For the E19C ensemble, dotted lines represent individual realizations, while thick continuous lines denote ensemble means. Brown squares at the bottom of panel (**c**) indicate months where the eVolv2k and eVolv2k-D4 ensembles differ significantly (*p* < 0.05) based on a Wilcoxon rank-sum test. Anomalies are calculated as monthly deviations from the 1780–1799 climatology for panels (**a**) and (**b**), and relative to the 1791–1810 period of the NoVolc control experiment for panel (**c**). Panel (**c**) redrawn from Fang et al. (2023)

The diversity in radiative forcing is, in turn, reflected in the spread of surface temperature responses across models and realizations. As shown by the anomalies in global-mean near-surface air temperature (Fig. 3), the divergent trajectories across models result from both disparate forcing and the unique feedbacks activated within each system.

Due to the specific thermodynamic and dynamical schemes of each model, there is no straightforward relationship between TOA radiative anomalies and global surface cooling—whether in terms of peak magnitude, timing, or the duration of the post-eruption recovery (e.g., Driscoll et al. 2012; Marshall et al. 2022). In the PMIP3 ensemble, the discrepancy between post-eruption TOA radiative anomalies and the resulting surface cooling in MPI-ESM-P and CCSM4 highlights fundamental differences in model sensitivities (Fig. 3a): CCSM4 exhibits stronger TOA radiative anomalies than MPI-ESM-P, yet it produces a notably weaker (and earlier peaking) surface cooling response. Differences are similarly striking in the PMIP4 ensemble, where models use the same eVolv2k reconstruction but different forcing implementations. Even when models produce similar TOA radiative anomalies—as seen in MIROC-ES2L and MRI-ESM2-0 (Fig. 2b)—their surface temperature responses can diverge significantly throughout the post-eruption period (Fig. 3b).

The absence of a one-to-one relationship between TOA radiative anomalies and surface cooling reflects intrinsic differences in climate sensitivity and feedbacks, as well as the disparate spatial and size distributions of the aerosols. Specifically, while MIROC-ES2L uses prescribed aerosol optical properties from EVA, MRI-ESM2-0 prognosticates aerosol evolution from precursors, leading to a fundamentally different physical implementation of the same eruptive event.

These apparently counterintuitive behaviors demonstrate that internal model characteristics—such as the climate feedback parameter, ocean heat uptake efficiency, or atmospheric rapid adjustments—can outweigh differences in the initial radiative forcing. Consequently, the TOA imbalance cannot be directly translated into the magnitude, timing, or duration of surface cooling. This underscores that the uncertainty in climate response is not simply inherited from forcing uncertainty, but also emerges from the diverse representation of Earth system processes within climate models (Table 1).

The puzzle, however, remains incomplete. The PMIP3 and PMIP4 multi-model ensembles discussed thus far in most cases consist of only a single realization per model, which fails to account for the dependence on initial conditions. The climate system—and by extension, its representation within Earth system models—is inherently nonlinear and chaotic. Consequently, a single simulation cannot be considered representative of the full range of possible forced responses, as internal climate variability can significantly mask or amplify the volcanic signal.

### The role of internal variability

The relative importance of internal variability increases markedly from the global to the regional scale. The E19C multi-forcing ensemble demonstrates that, when a specific forcing is imposed, it exerts tight constraints on the evolution of global-mean temperatures. This is evident in the individual realizations, which remain closely clustered around the ensemble mean for each of the eVolv2k and eVolv2k-D4 experiments (Fig. 3c). Such reduced internal spread allows for the identification of subtle emerging signals: for instance, small-to-moderate eruptions occurring in the aftermath of the 1809 and 1815 events produce a statistically significant separation between the eVolv2k and the eVolv2k-D4 ensemble means (brown squares in Fig. 3c). Consequently, the inclusion of these secondary volcanic events results in a protracted surface temperature recovery, shifting the transition back to baseline conditions.

**Fig. 4:**
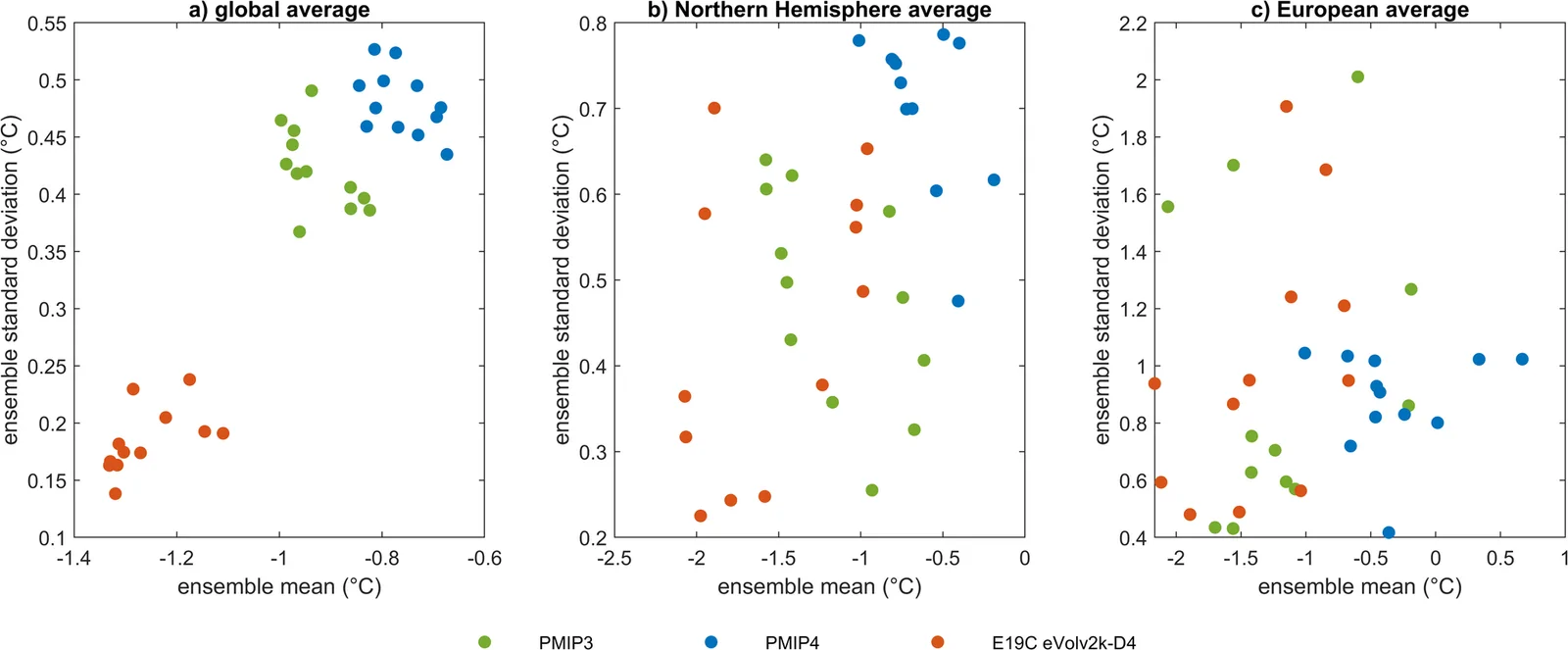
Scaling of intrinsic climate variability across spatial domains. Scatterplots of ensemble means versus ensemble standard deviations for the PMIP3, PMIP4, and E19C eVolv2k-D4 ensembles. Data represent monthly 2-meter air temperature (*tas*) anomalies for the year 1816, spatially averaged over (**a**) the globe, (**b**) the Northern Hemisphere, and (**c**) Europe. Anomalies are calculated relative to the climatologies defined in Fig. 3. The increasing spread relative to the mean signal from panel (**a**) to (**c**) illustrates the growing dominance of internal variability at finer spatial scales

However, internal variability plays an increasingly dominant role in broadening the response as smaller spatial domains are considered. This is clearly illustrated in Fig. 4, which compares the ensemble mean and spread for post-Tambora anomalies across three datasets—the multi-model PMIP3 and PMIP4 ensembles, and the single-model eVolv2k-D4 ensemble from the E19C multi-forcing ensemble—at global, hemispheric, and regional scales.

At the global scale, the spread driven by model diversity is two to three times larger than that generated by internal variability (Fig. 4a). The high standard deviation and relatively small mean anomaly in the PMIP4 ensemble stem largely from the inclusion of the ACCESS-ESM1-5 model, which exhibits virtually no TOA radiative flux perturbations, hence no post-Tambora cooling (Fig. 3b). Nonetheless, the clear clustering of ensemble mean and spread suggests that at the global scale, the direct radiative response predominates, reflecting model-specific factors such as forcing implementation, climate sensitivity, and ocean heat uptake.

Taken together, these results show that the climate response to volcanic forcing is not uniquely determined by the forcing itself, but emerges from the interplay between forcing characteristics, internal variability, and state-dependent feedbacks.

Moving to the hemispheric scale, the distinction between the ensemble scatterplots becomes less defined. The spread of the single-model eVolv2k-D4 ensemble largely overlaps with the multi-model PMIP3 ensemble, despite the former producing significantly stronger cooling (Fig. 4b). At the regional scale—exemplified here by European mean temperatures—the single-model ensemble produces a spread comparable to both multi-model ensembles (Fig. 4c). At this spatial resolution, the response becomes less dependent on direct radiative effects as the dynamical component predominates. This highlights the challenge of constraining circulation changes, which depend not only on the volcanic forcing but also on the initial state of the system, i.e., the “butterfly effect”.

The increasing importance of internal variability at regional scales raises a broader question: how does it compare with other sources of uncertainty, such as eruption magnitude, location, forcing history, and model structure? To address this question, we apply a k-means clustering method following Zanchettin et al. (2019).

This approach is based on the premise that the Tambora, E19C, EVA40, and *volc-long-eq* super-ensembles each comprise three distinct sub-ensembles defined by their experimental design (e.g., varying forcing levels or spatial structures, or different models). By feeding each super-ensemble into a k-means algorithm with k = 3, we test whether the design factor is the dominant source of uncertainty: if so, the resulting clusters should largely coincide with the original sub-ensembles. Conversely, if clusters are heterogeneous, this indicates that internal variability or model diversity outweighs the experimental forcing differences.

The clustering is intended as an exploratory diagnostic rather than a formal variance decomposition. Because each super-ensemble samples a different combination of forcing uncertainty, model structure, and internal variability, the resulting clusters cannot be directly compared across experiments. Instead, they provide a process-oriented assessment of whether the imposed experimental design emerges over background variability within each ensemble.

**Fig. 5:**
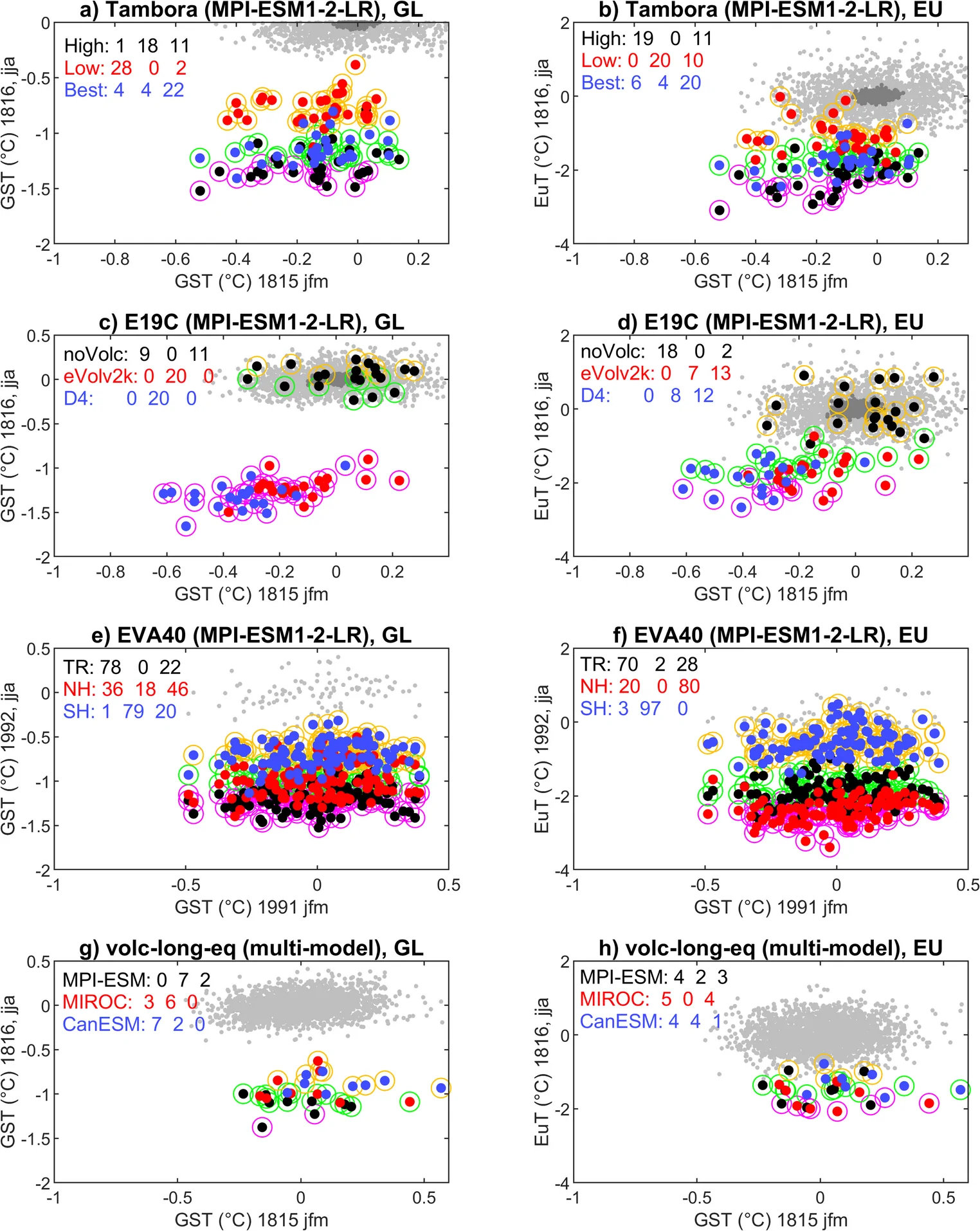
Decomposition of uncertainties in the post-Tambora climate response. Comparison of initial condition unknowns versus other sources of uncertainty for the global (GL; left panels) and European (EU; right panels) domains. The rows illustrate different uncertainty drivers: (**a**, **b**) sulfur emission magnitude (Tambora ensemble); (**c**, **d**) influence of background volcanic activity (inclusion/exclusion of small-to-moderate eruptions, E19C ensemble); (**e**, **f**) spatial structure of the forcing (EVA40 ensemble); and (**g**, **h**) model-specific responses (*volc-long-eq* ensemble). Original ensemble members are represented by small filled circles (blue, red, black), while orange, green, and magenta large circles denote the k-means clusters. Grey and dark grey dots represent the range of intrinsic variability derived from *piControl* (**a**–**d**, **g**, **h**) and eva0 (**e**, **f**) simulations. Numbers in the top-left corners indicate the distribution of original ensemble members across the three k-means clusters. Panels (**a**, **b**) are adapted from Zanchettin et al. (2019), Fig. 2 (a, e). Panels (a, b) redrawn from Zanchettin et al. (2019)

We applied this method to summer (JJA) mean temperatures for 1816, averaged over the globe and Europe. As shown in Fig. 5, for global-mean surface temperatures, the magnitude (Tambora ensemble, panel a), location (EVA40 ensemble, panel e), and model differences (*volc-long-eq* ensemble, panel g) strongly constrain the post-eruption cooling. In these cases, realizations from each sub-ensemble primarily populate a single k-means cluster. Nevertheless, the influence of secondary uncertainties is still detectable, with approximately 20% of ensemble members falling into secondary clusters.

The E19C ensemble (panel c) exhibits a peculiar behavior: the algorithm groups all realizations from the eVolv2k and eVolv2k-D4 ensembles into a single cluster, while partitioning the NoVolc ensemble into the remaining two. This suggests that, from the blind perspective of the clustering algorithm, unforced internal variability is more statistically separable than the differences between different volcanic forcing histories in the aftermath of 1815.

The influence of experimental design is notably more subtle for European temperatures. While post-eruption cooling remains a common feature across all ensembles, the resulting clusters exhibit a much higher degree of mixing between the original sub-ensembles (Fig. 5b, d, f, h). In this regional context, the primary cluster typically accounts for only 40% to 60% of the original members, as post-eruption anomalies shift closer to the range of intrinsic variability compared to the more distinct global-mean responses. This highlights the dominant role of internal variability in shaping the spread of regional climate responses, particularly at smaller spatial scales.

The EVA40 ensemble provides a clear illustration of this spatial dependency: for a Southern Hemisphere eruption, European cooling largely overlaps with unforced internal variability. In contrast, the most intense European cooling is triggered by a Northern Hemisphere eruption, whereas the maximum global-mean cooling is associated with a tropical eruption (Fig. 5e, f).

Finally, the *volc-long-eq* ensemble shows the most uniform distribution across the three clusters (Fig. 5h). This lack of separation may suggest a certain robustness in regional model responses, but it also underscores the statistical limitations inherent in the small ensemble sizes of the *volc-long-eq* protocol, which may be insufficient to fully disentangle model-specific signals from regional noise.

Taken together, these contrasting clustering behaviors show that the apparent importance of individual uncertainty sources depends strongly on experimental design. Because forcing uncertainty, model structure, and internal variability are fundamentally intertwined, their individual contributions cannot be cleanly isolated through cross-ensemble statistical comparisons. This motivates the process-oriented analysis of winter dynamics that follows.

### Physical mechanisms of state-dependent responses

Whereas summer responses are dominated by direct radiative effects, winter responses are increasingly shaped by coupled atmosphere-ocean dynamics, making post-eruption winter climate variability substantially more state dependent (Zanchettin et al., 2019). In the extratropics, post-eruption circulation changes are primarily influenced by the so-called stratospheric pathway. This top-down mechanism is triggered by the absorption of terrestrial and near-infrared radiation by sulfate aerosols, which warms the tropical lower stratosphere, thereby steepening the equator-to-pole temperature gradient. Through thermal wind balance, this strengthened gradient can intensify the winter stratospheric polar vortex, a signal that often propagates downward to favor a positive phase of the North Atlantic Oscillation (NAO). Historically, this process has been invoked to explain the “volcanic winter warming” pattern characterized by strengthened westerlies and higher temperatures over Northern Eurasia.

Recent studies, however, indicate that this dynamical response may be far less deterministic than previously assumed. While stratospheric warming remains a robust feature, its surface expression is frequently masked by intrinsic atmospheric variability. Ensemble simulations demonstrate that even when a positive NAO emerges in the ensemble mean, individual realizations exhibit a broad spectrum of responses, including anomalies of opposite sign (e.g., Polvani and Camargo 2020; DallaSanta and Polvani 2022). As observed for summer responses, this diversity is compounded by the background climate state—particularly the phase of the Quasi-Biennial Oscillation (QBO), as extensively documented in literature (e.g., Thomas et al. 2009; Toohey et al. 2014), and the seasonal timing of the eruption—highlighting that the stratospheric pathway is a conditional rather than a guaranteed driver of post-eruption regional climate anomalies.

Uncertainty surrounding the stratospheric pathway and the associated “volcanic winter warming” is further compounded by the tropical response. Disentangling the shortwave and longwave radiative forcing components reveals that they exert independent and fundamentally different dynamical influences on the zonal asymmetries of extratropical Northern Hemisphere winter temperatures. Whereas the shortwave forcing (i.e., albedo increase) induces a tropospheric Rossby wave train seemingly connected to perturbations in tropical convection, the long-wave properties (i.e., stratospheric aerosol heating) generate a zonal wavenumber-1 signal in surface temperatures, which is directly related to the strengthening of the stratospheric polar vortex and altered zonal winds (Andreasen et al. 2026). Furthermore, the tropical Pacific state can act as a critical modulator: the El Niño-Southern Oscillation (ENSO) can exert a modulating influence on the strength of the polar vortex and the NAO (e.g., Graf et al. 2014). Although several modeling and proxy-based studies suggest that large tropical eruptions may favor or trigger El Niño–like conditions (e.g., Pausata et al. 2015), this response remains highly uncertain and model-dependent (D’Agostino et al. 2026). This lack of consensus likely stems from the confounding effects of internal variability, eruption timing, and uncertainties in proxy sensitivity or dating. Consequently, the prevailing view suggests that volcanic forcing may modulate the probability distribution of ENSO states rather than consistently triggering a canonical event, further emphasizing the critical role of the pre-eruption background state.

The North Atlantic provides a clear example of how rapid atmospheric adjustments interact with slower oceanic feedbacks, introducing an additional layer of memory and state dependence. A stratospheric-driven NAO phase can theoretically accelerate the Atlantic Meridional Overturning Circulation (AMOC)—a critical component of poleward oceanic mass and heat transport—through enhanced buoyancy loss in the Labrador and Greenland–Iceland–Norwegian Seas. However, the robustness of this link is undermined by the chaotic nature of the atmosphere and by the propagation of model-specific differences related to the forcing and the background climate conditions. The inherent uncertainty in the NAO response—often indistinguishable from internal noise for a Pinatubo-size eruption (Zanchettin et al. 2022)—is further amplified by the ocean’s diverse sensitivity to pre-eruption conditions. Discrepancies among models regarding the role of sea-ice feedbacks and freshwater fluxes (Zanchettin et al. 2012; Swingedouw et al. 2015) lead to a substantial spread in simulated AMOC anomalies (Ding et al. 2014; Zanchettin et al. 2016).

**Fig. 6:**
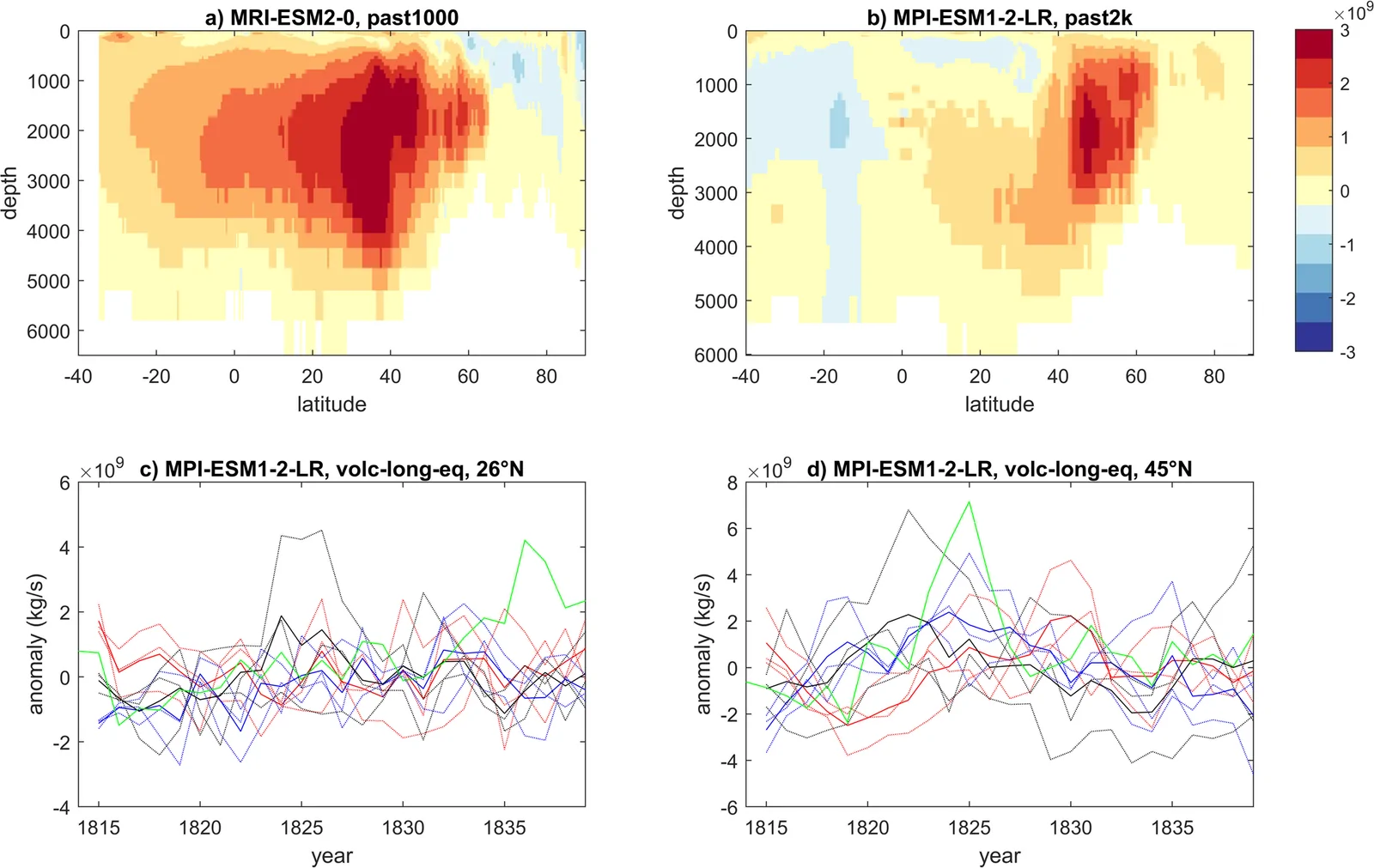
Model and initial condition dependency of the post-Tambora AMOC evolution. (**a**, **b**) Spatial difference in the annual-average ocean meridional overturning mass streamfunction between the post-eruption (1820–1825) and pre-eruption (1790–1800) periods for (**a**) MRI-ESM2-0 (*past1000*) and (**b**) MPI-ESM1-2-LR (*past2k*). (**c**, **d**) Evolution of annual AMOC indices for (**c**) the tropical (26°N, 1000 m depth) and (**d**) the mid-latitude (45°N, 2000 m depth) bands from the MPI-ESM1-2-LR *volc-long-eq* ensemble. Indices are shown as anomalies relative to the *piControl* climatology and categorized by the initial AMOC state: strong (red), neutral (black), and weak (blue). The green line represents the corresponding AMOC index from the *past2k* simulation

Model-specific differences are strikingly apparent in the spatial representation of oceanic responses (Fig. 6a, b). While both MPI-ESM1-2-LR and MRI-ESM2-0 simulate a post-Tambora strengthening of the meridional overturning mass streamfunction, their patterns diverge appreciably: the response in MPI-ESM1-2-LR is confined to midlatitudes at depth, whereas MRI-ESM2-0 produces a basin-wide, vertically extended intensification.

Beyond inter-model diversity, the supervised sampling of initial conditions in the *volc-long-eq* experiment reveals a critical source of intra-model spread. As illustrated in Fig. 6b and c, the AMOC response in MPI-ESM1-2-LR is highly sensitive to the pre-eruption state. Notably, a strong pre-eruption AMOC phase tends to mute the volcanic response, whereas a weak or neutral state facilitates a more pronounced post-eruption strengthening. Although the anomaly also depends on the reference period, this enduring ocean dependence—first identified in pre-PMIP3 ensembles (Zanchettin et al. 2012)—is particularly relevant for volcanic clusters, such as the early-nineteenth-century sequence. When the interval between eruptions is shorter than the characteristic timescale of the AMOC response, subsequent eruptions become tightly dependent events. Their effects can superpose constructively or destructively depending on the precise timing and the phase of the ocean at the moment of the second injection (Swingedouw et al. 2015), highlighting the potential for nonlinear interference between successive eruptions. Therefore, the decadal to multidecadal AMOC response to an eruption depends not only on the eruption itself, but on the mean climate state, the volcanic history, and the presence and intensity of other concomitant forcing factors (Zanchettin et al. 2013; Fang et al. 2021; Dutta et al. 2025).

Overall, this oceanic memory allows the system to integrate and prolong volcanic cooling for decades. However, its surface expression remains governed by a complex interplay of forcing characteristics, model-specific physics, and initial conditions—such as, for Europe, the background stratification of the North Atlantic subpolar gyre and the Arctic sea-ice extent. Consequently, the regional climate response is not a simple linear reflection of radiative forcing, but an emergent property of a coupled, nonlinear system, in which intrinsic variability can mask or fundamentally reshape the deterministic volcanic signal.

In light of these multi-layered uncertainties, a fundamental question emerges: how can we rigorously compare climate model ensembles with observational or reconstruction targets to interpret regional variability after specific events? This challenge forms the core of the following section on fingerprinting and attribution.

## Fingerprinting and attribution

In the context of detection and attribution of climate change, fingerprinting refers to the statistical technique of isolating the unique spatial and temporal patterns of climate response—such as specific cooling gradients or circulation changes—that distinguish the forced signal from the noise of intrinsic variability. In the case of volcanic forcing, fingerprinting therefore allows the quantification of the contribution of volcanic aerosols to observed climate anomalies by matching simulated response patterns against instrumental or proxy-based reconstructions (e.g., Hegerl and Zwiers 2011).

**Fig. 7:**
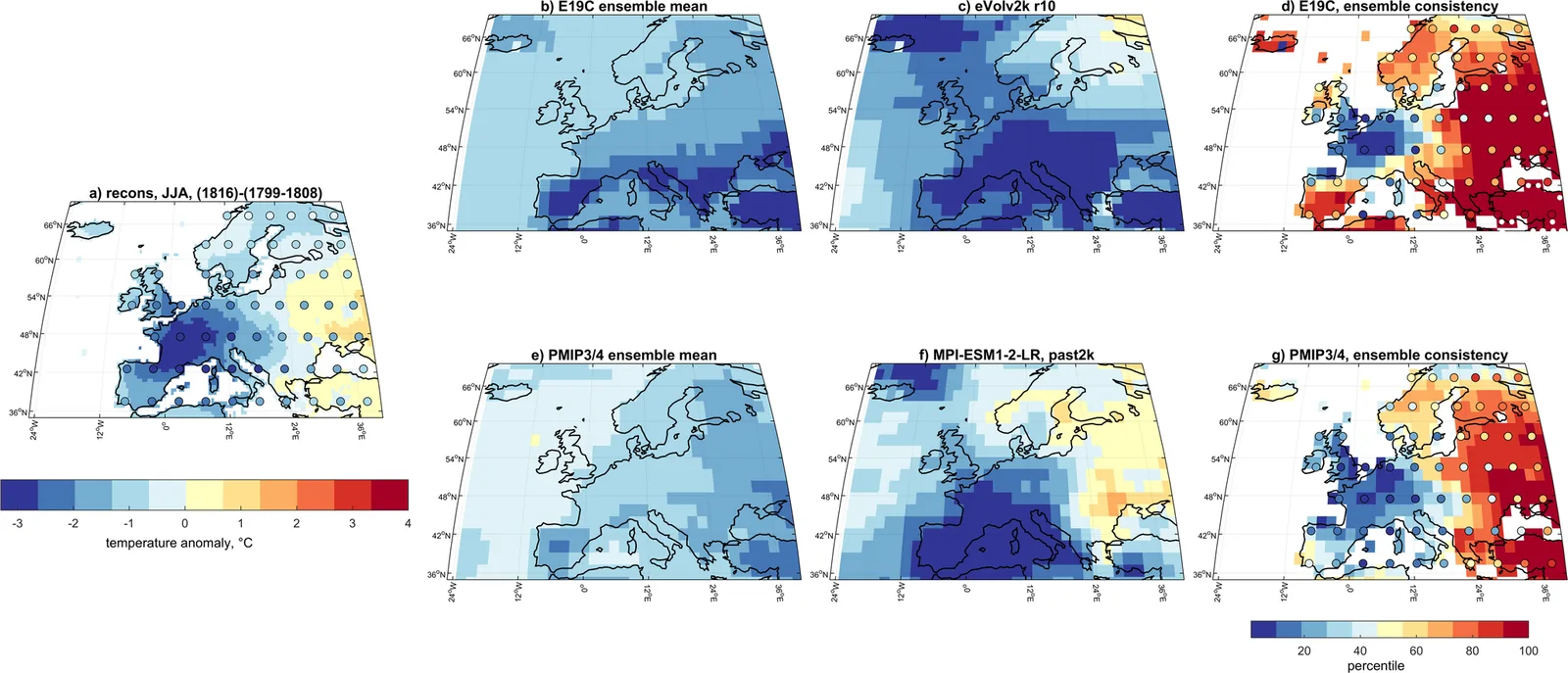
Reconstructed versus simulated European temperature patterns for the 1816 “Year Without a Summer”. (**a**) Reconstructed JJA temperature anomalies (shading: Luterbacher et al. 2004; points: EuroMed2k summer temperature reconstruction, Luterbacher et al. 2016). (**b**–**d**) Results from the combined E19C ensemble (eVolv2k and eVolv2k-D4) and (**e**–**g**) the combined PMIP3 and PMIP4 multi-model ensembles. The panels illustrate: (**b**, **e**) ensemble means; (**c**, **f**) a representative simulation showing high spatial correlation with the reconstructions; and (**d**, **g**) an assessment of ensemble consistency. Consistency is shown as local percentiles of the reconstruction relative to the ensemble distribution evaluated for both targets (shading: Luterbacher et al. 2004; points: EuroMed2k summer temperature reconstruction). For Luterbacher et al. (2004), white dots identify locations where the reconstructed data is an outlier (*p* < 0.05 or *p* > 0.95) relative to the simulations. All anomalies are JJA 1816 deviations from the 1799–1808 seasonal climatology

The 1816 “Year Without a Summer” in Europe, characterized by anomalous cold, unusual wetness, and subsequent agricultural failure, serves as an iconic case study for volcanic impacts on regional climate. Figure 7a illustrates the summer (JJA) temperature anomalies from two complementary paleoclimatic products: the spatial reconstruction by Luterbacher et al. (2004) and the updated EuroMed2k gridded dataset (Luterbacher et al. 2016). While both sources agree that severe cooling was concentrated over Central Europe—which diminishes toward the Mediterranean, the British Isles, and Scandinavia—they exhibit a stark structural divergence over Eastern Europe. In this eastern sector, the 2004 reconstruction suggests a prominent warm anomaly, whereas the 2016 EuroMed2k data indicate a cold anomaly, highlighting that regional paleoclimatic targets carry non-uniform spatial uncertainties.

In light of these observational uncertainties, shifting the focus from purely thermodynamic variables (like surface temperature) to large-scale atmospheric circulation dynamics becomes essential for a robust regional attribution. Recent event attribution studies of 1816 underscore the critical necessity of disentangling radiative forcing from dynamical variability. By employing an analogue-based approach built on PMIP4-class simulations to isolate the role of atmospheric circulation, Schurer et al. (2019) demonstrate that while the volcanic forcing from Tambora made the European cold anomaly up to 100 times more likely, the circulation state alone could reproduce the observed precipitation patterns even without external forcing. This confirms that when spatial proxy targets conflict or show localized uncertainties, linking climate anomalies to large-scale circulation diagnostics provides an indispensable physically consistent framework. Without anchoring the analysis to this dynamical context, the forced volcanic signal risks being either misattributed due to proxy noise or conflated with stochastic regional weather variability.

The potential divergence between independent reconstructions, paired with the challenge of separating forced from intrinsic dynamics, directly illustrates the complex propagation of uncertainties from the global volcanic source to the regional climate response (Table 1). Rather than a simple, linear alignment between theory and observation, regional attribution emerges as a non-linear problem where uncertainties in both the historical proxy targets and model physics amplify as we downscale from global radiative forcing to local circulation anomalies.

However, we now pivot to a different perspective: is the historical “Year Without a Summer” a representative realization of what we should structurally expect from a Tambora-like eruption? In other words, how robustly can modern climate models simulate this complex, reconstructed regional variability, when observational targets themselves are uncertain, and what are the most critical modeling advancements needed to reproduce or bound these patterns?

To address this question, we analyze the combined eVolv2k and eVolv2k-D4 ensembles from the E19C super-ensemble alongside the combined PMIP3/4 multi-model ensembles. Rather than attempting a formal, fully coupled propagation of reconstruction errors, we adopt a pragmatic approach tailored for illustrative perspective: we evaluate simulation fidelity against each of the two available paleoclimatic reconstructions independently. By treating Luterbacher et al. (2004) and the EuroMed2k as distinct, plausible observational targets, we can qualitatively bound the historical target uncertainty and evaluate how the choice of reconstruction alters our assessment of ensemble consistency.

The ensemble means reveal divergent systematic features: the E19C super-ensemble produces a meridionally asymmetric cooling pattern with the most intense cooling over Mediterranean Europe (Fig. 7b), whereas the PMIP3/4 super-ensemble yields a more spatially uniform cooling, in which both model-specific nuances and circulation-driven anomalies are averaged out (Fig. 7e). Because averaging across realizations emphasizes the direct radiative response while dampening stochastic dynamical effects, neither ensemble mean successfully reproduces the full regional complexity found across the reconstructions (Fig. 7b, e). Specifically, the ensemble means fail to capture the intense, localized peak cooling over Central Europe prominent in both proxy targets. Furthermore, they do not consistently replicate the zonal asymmetry of Luterbacher et al. (2004) characterized by warmth over Eastern Europe.

Instead, when evaluated against the EuroMed2k data, only the PMIP3/4 ensemble mean aligns with the rather uniform cooling across latitudes in Eastern Europe. Conversely, only E19C partially captures the reconstructed central Mediterranean cooling evident in the EuroMed2k marine and coastal data points over the Tyrrhenian and Adriatic sectors (Fig. 7a). Summer cooling over the Mediterranean emerges as a consistent dynamical fingerprint in multi-model analyses of major volcanic eruptions throughout the last-millennium; this is primarily driven by a systematic alteration of the prevailing surface wind patterns and a reduction in south-westerly moisture advection (Misios et al. 2022). Whereas MPI-ESM1-2-LR apparently captures this dynamical feature, its absence in the PMIP3/4 multi-model mean may stem from either a lack of a robustly forced dynamic component across the broader suite of models or a masking effect induced by a biased, overly uniform thermodynamic response.

These results underscore that ensemble means possess limited predictive power when measured against a specific historical target like the “Year Without a Summer”. Whereas in some sectors, such as Scandinavia, a broad alignment across both reconstructions and ensembles points toward a predictable, thermodynamically forced cooling, Central Europe tells a different story. As the key defining feature of the “Year Without a Summer”, Central Europe experiences less than half of the reconstructed cooling in both ensemble means. Barring structural model biases in representing regional atmospheric dynamics, this discrepancy suggests that the historical 1816 event was not a direct, linear representation of the volcanically forced mean state. Instead, it was likely an extreme manifestation of internal variability combined with the volcanic cooling signal.

This realization necessitates a probabilistic assessment of consistency to determine whether the reconstructed climate targets lie within the range of simulated behaviors, highlighting that deterministic comparisons against single or a few realizations can be fundamentally misleading for regional fingerprinting and attribution. We quantify this by mapping the local rank of each reconstruction as a percentile against the ensemble distribution (Fig. 7d, g). In this framework, dark blue and dark red areas identify regions where a reconstruction falls into the extreme lower or upper tails of the ensemble spread, respectively, signaling potential local inconsistencies between the model outputs and the proxy-based targets.

Despite their different experimental designs, both percentile maps reveal how the assessment of ensemble consistency shifts depending on the choice of proxy target (Fig. 7d, g). When evaluated against the Luterbacher et al. (2004) baseline, both ensembles exhibit a similar zonal dipole of opposing discrepancies, where the simulations are systematically warmer than the reconstruction over Central Europe (blue tails) while remaining cooler over Eastern Europe (red tails). Crucially, however, using the EuroMed2k data substantially mitigates this eastern inconsistency. Over Eastern Europe, the extreme red tails largely disappear, replaced by widespread yellow and orange pixels that denote historical anomalies well within the internal variability of both the E19C and PMIP3/4 distributions. This demonstrates that the previously diagnosed eastern discrepancy was primarily an artifact of reconstruction uncertainty rather than a fundamental model failure.

In contrast, the warm bias over Central Europe remains a robust, shared feature across both ensembles regardless of the paleoclimatic target used. The persistence of this Central European offset across both a single-model large ensemble (E19C) and a multi-model framework (PMIP) indicates that even when accounting for the full range of simulated evolutions, these models systematically struggle to capture the intense, localized dynamical signature of the historical anomaly. This underscores that while updating proxy targets can successfully reconcile apparent model-data conflicts in some domains, the extreme cooling of Central Europe in 1816 remains a fundamental challenge for CMIP- and PMIP-class models.

Interestingly, while several members of the E19C ensemble show a generalized European cooling, such as the widespread cold anomaly depicted in Fig. 7c, MPI-ESM1-2-LR is also dynamically capable of simulating a zonal dipole like the one exhibited by the Luterbacher et al. (2004) reconstruction. This is shown, as an example, by the MPI-ESM1-2-LR *past2k* simulation (Fig. 7f), but also emerges in at least one individual E19C member (r17 of eVolv2k-D4, not shown). Given that this zonal dipole is not robust across reconstructions and may not represent an accurate historical target, the physical significance of finding a strong spatial match diminishes. Nevertheless, this overlapping result underscores that identifying a simulation that captures a specific proxy target is largely a function of sampling density and ensemble size. Relying on a limited number of realizations per model prevents a robust assessment of each model’s true internal range of behaviors. Without large ensembles to sufficiently map the low-probability tails of regional atmospheric dynamics, it remains fundamentally impossible to distinguish between a model’s structural inability to simulate the 1816 anomaly and a simple under-sampling of its internal variability continuum.

To better understand this structural diversity and explore the physical drivers of these mismatched regional responses, we calculate the summer (JJA) SNAO index for the PMIP3/4 ensemble using surface pressure data (UKESM1 excluded because the required surface pressure fields were unavailable), and for the E19C large ensemble using mean sea level pressure data, applying the box-based method (Zanchettin et al. 2016). To facilitate comparison between models and reconstructions, all summer 1816 anomalies are standardized relative to the 1799–1808 climatology.

The resulting standardized anomalies indicate that the large uncertainty in simulated surface temperatures is consistent with a strong role of internal atmospheric variability. Even historical reconstructions (Luterbacher et al. 2001; Folland et al. 2009) do not offer a consensus on the dynamical state of that summer, with different datasets showing opposite signs—ranging from a slight positive phase to a negative anomaly exceeding one standard deviation. The PMIP3 and PMIP4 models broadly mirror this deep uncertainty. While the PMIP3 ensemble exhibits a general tendency toward negative SNAO anomalies, with some members plunging well below two to three standard deviations, the PMIP4 simulations are highly divergent. Within PMIP4, individual responses span from moderately positive phases to weak negative anomalies close to neutrality, which is where MPI-ESM1-2-LR places its response. This dynamical behavior of MPI-ESM1-2-LR is further clarified by the E19C ensemble: while NoVolc members cluster around neutral-to-weak anomalies, the inclusion of volcanic forcing (both eVolv2k and eVolv2k-D4) induces a systematic shift toward negative SNAO phases, reproducing extreme negative anomalies down to over three to four standard deviations, while still retaining a large spread driven by internal variability. This diversity in atmospheric circulation regimes is clearly reflected in the temperature anomalies: the widespread cooling in eVolv2k r10 (Fig. 7c) occurs under a nearly neutral SNAO state, suggesting a predominantly radiatively driven response, whereas the dipole pattern in the eVolv2k-D4 r17 is accompanied by a strong negative SNAO anomaly exciding 2σ in magnitude, highlighting the crucial role of atmospheric dynamics.

This wide spread indicates that the regional climate footprint of the 1816 “Year Without a Summer” depended strongly on the atmospheric circulation state, rather than being predominantly determined by the direct radiative response alone.

Taken together, these findings suggest two complementary interpretations of the 1816 European summer. Either current Earth system models systematically underestimate the forced circulation anomalies or local feedbacks required to reproduce the reconstructed regional pattern, or the historical event reflects an exceptionally unusual state of the internal atmospheric circulation. In the latter case, reproducing the observed anomaly requires ensembles large enough to sample the far tails of internal climate variability, where statistically rare yet physically plausible realizations reside.

Ultimately, this dual perspective underscores that the volcano–climate puzzle remains fundamentally incomplete without a probabilistic framework. Large-ensemble experiments—such as EVA40 and E19C—are indispensable not only to distinguish between fundamental model deficiencies and the inherent unpredictability of regional extremes but also to map out the continuum of simulated behaviors against which structurally uncertain paleoclimatic targets can be rigorously evaluated.

## Ways forward: reframing the volcano–climate puzzle

Volcanism is one of the most influential natural forcing agents of climate variability across historical and pre-industrial periods, and it remains an important source of uncertainty for near-future climate projections. While the fundamental principles of volcano–climate interactions—from aerosol–radiation physics to global-scale thermodynamic responses—are now well-established, this Perspective has highlighted the persistent difficulty of constraining these interactions for specific events outside the instrumental era.

**Fig. 8:**
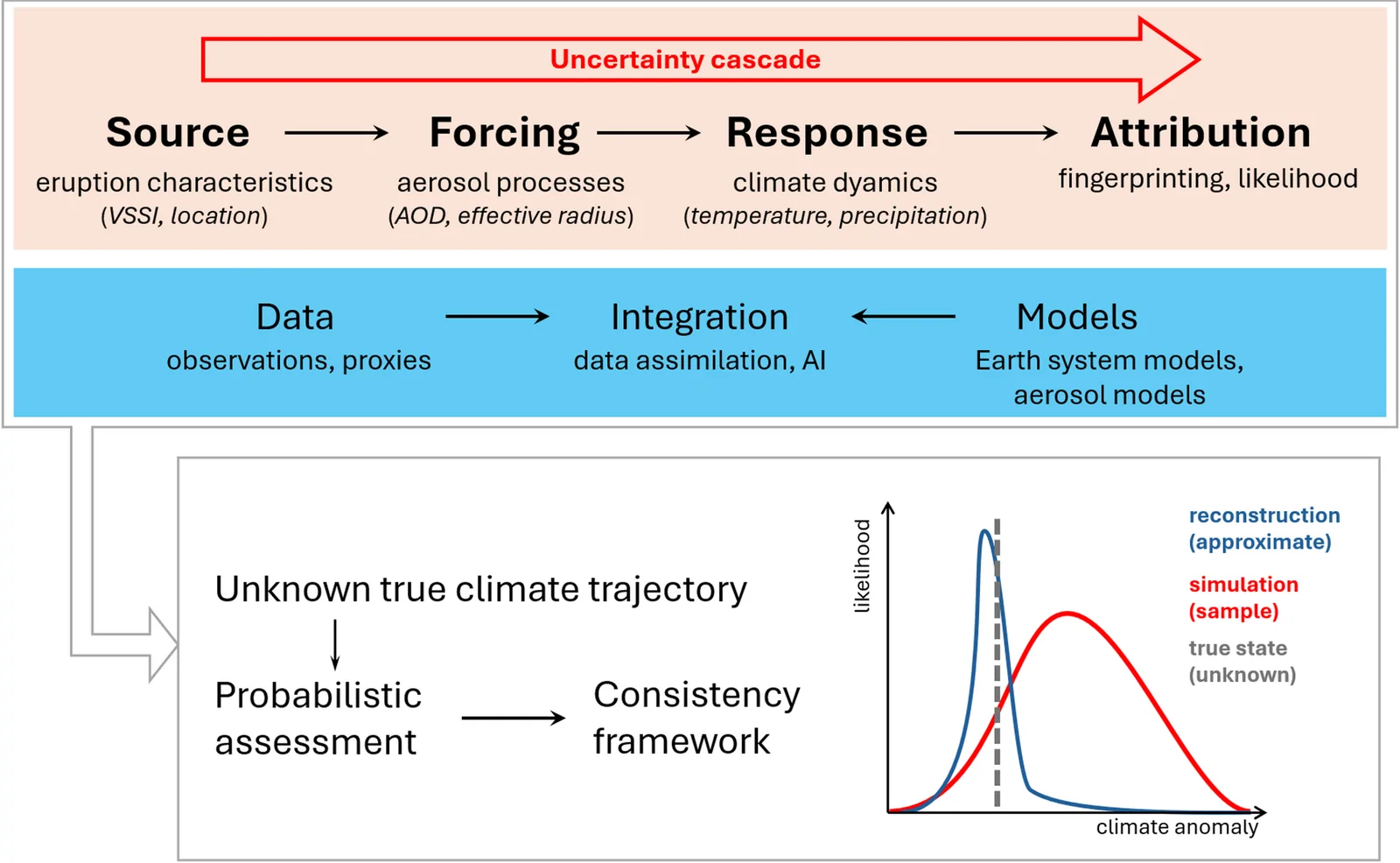
Conceptual framework of the volcano–climate puzzle for pre-observational eruptions. The schematic illustrates the propagation of volcanic signals across the coupled Earth system—from eruption source characteristics through radiative forcing and climate response to fingerprinting and attribution—highlighting the associated cascade of uncertainties (red box). The central pathway emphasizes how uncertainties originating in source characterization propagate through aerosol processes and climate dynamics, ultimately affecting the attribution of regional climate anomalies. The blue box represents the integration of observational data (e.g., proxies and documentary evidence) and modeling approaches (e.g., Earth system models, data assimilation, and AI-based tools) to constrain an uncertain real-world climate trajectory. The bottom section frames the probabilistic assessment in terms of consistency between reconstructed and simulated climate anomalies. The probability density functions illustrate the distributions of reconstructions (blue) and model simulations (red), while the vertical dashed line denotes the unknown true climate state. The degree of overlap between these distributions reflects the likelihood that reconstructed anomalies are consistent with the ensemble of simulated outcomes, given uncertainties in volcanic forcing, initial conditions, and model representation

The uncertainty cascade examined here, spanning volcanic source characterization, forcing generation, radiative and dynamical climate responses, initial-condition dependence, and attribution, shows that even relatively recent events such as the early-nineteenth-century volcanic cluster remain only partially constrained (Fig. 8). Framed as an operational sequence (Table 1), the main bottleneck is no longer the understanding of individual physical components, but rather our ability to constrain how uncertainties propagate across them, from VSSI estimates to the probabilistic attribution of regional anomalies such as the 1816 European summer.

Because these uncertainties are physically intertwined and cannot be cleanly separated through classical variance decomposition, future progress will require explicit probabilistic approaches. Treating forcing reconstructions as prior distributions and using multi-model large ensembles to constrain the conditional likelihood of atmospheric regimes offer a rigorous way to update interpretations of past events without artificially decoupling processes that are physically entangled.

The recognition that uncertainty is not merely a limitation but an enduring feature of volcano–climate research recalls Socrates’ paradox—that true wisdom lies in recognizing the vastness of what we do not yet know. Rather than representing obstacles to progress, the uncertainties highlighted throughout this Perspective define the key scientific frontiers of the field. Together, they call for a fundamental shift in how volcanic climate impacts are reconstructed, simulated, and interpreted. Accordingly, we identify five trajectories to move the volcano–climate puzzle forward. These trajectories span methodological, technological, and conceptual advances.

### Methodological reframing: forward and backward approaches

To systematically disentangle the uncertainty cascade, future research must adopt a dual-track analytical approach, combining process-based understanding with constraint-based inference. First, future studies should adopt a mechanism-centric forward approach that isolates the cascade of interactions linking eruption source, aerosol microphysics, radiative forcing, and climate response. Multi-model assessments under idealized and realistic conditions—such as those conducted under ISAMIP (Timmreck et al. 2018), VolMIP (Zanchettin et al. 2016; Marshall et al. 2018; Clyne et al. 2021) or DCPP (Bilbao et al. 2024; Sospedra-Alfonso et al. 2024)—are essential. Evidence from the VolMIP *volc-pinatubo-full* experiment indicates that model diversity is often confounded by initial condition sampling and mean biases (Zanchettin et al., 2022). Future studies should increasingly exploit stronger forcing, such as Tambora-like eruptions, to better isolate deterministic mechanisms from internal variability. In this context, the development of systematic diagnostics—such as scatterplots comparing TOA peak forcing against peak global-mean surface temperature anomalies or regional recovery times—will be essential tools to rigorously map and constrain the structural diversity of model responses along the forcing-response cascade.

Second, a complementary constraint-centric backward approach can identify regional responses that are inconsistent with specific forcing scenarios. For the 1809 eruption, for instance, backtracking has shown that multiple combinations of magnitude and distribution could yield similar global temperatures, demonstrating that proxy data alone cannot uniquely constrain volcanic forcing (Timmreck et al. 2021). Using reconstructions as a filter rather than a target allows us to reject incompatible forcing estimates.

In parallel, continued improvements in volcanic forcing reconstructions remain essential. Future reconstructions should become increasingly spatially and temporally resolved, incorporating clustered small-to-moderate eruptions, which we have shown contribute meaningfully to decadal variability (Fang et al. 2023). They should also better account for the nonlinear behavior of aerosol microphysics, as rapid particle growth and enhanced sedimentation reduce the radiative efficiency of very large sulfur injections (Timmreck 2012).

Advances in reduced-complexity aerosol emulators provide an important opportunity to address these challenges. While simplified scaling approaches have traditionally struggled to represent the nonlinear dependence of forcing on injection height, aerosol microphysics, and background climate conditions, new-generation frameworks such as EVA_H (Aubry et al. 2020) substantially narrow the gap with fully interactive aerosol–climate models. By explicitly representing the vertical structure of sulfate aerosol forcing and plume dynamics, these emulators offer a robust and computationally efficient framework for large-ensemble climate simulations and probabilistic assessments of volcanic forcing.

### The next-generation toolbox: resolution and emulation

A recurring theme in our analysis is the potential discrepancy between the coarse resolution of PMIP- and CMIP-class models and the fine-scale nature of regional climate anomalies. Our failure to capture specific features, such as the 1816 summer zonal dipole, suggests that the representation of regional feedbacks remains a significant bottleneck. We foresee two ways forward.

First, future studies should increasingly exploit convective-permitting and high-resolution global or regional models to properly simulate local-scale processes. PMIP-class models have traditionally remained confined to low-resolution versions due to the extreme computational demands of millennial-scale simulations (e.g., Jungclaus et al. 2017). With a few notable exceptions (e.g., Brohan P et al. (2016); Hartman et al. 2025) there has been limited use of regional downscaling in the field of paleoclimatology and the study of volcanic impacts on climate. Expanding this approach would allow the community to bridge the gap between large-scale response pathways and specific local impacts. Furthermore, modern architectures like ICON offer the possibility of seamless transitions between global and local scales (Zängl et al. 2015; Jungclaus et al. 2022). These are therefore likely to become a key tool for future volcano–climate experiments, with dedicated designs exploiting scalability to capture critical land-surface interactions, soil-moisture feedbacks, and the interaction between orography and post-eruption circulation changes—all of which are likely underestimated in coarse-gridded general circulation and Earth system models.

Second, a critical frontier lies in the integration of emulators and Artificial Intelligence (AI)-based methods. Given the prohibitive computational cost of high-resolution ensembles, AI-driven emulators can serve as efficient surrogates for Earth system models, allowing systematic exploration of the vast parameter space defined by eruption characteristics, forcing scenarios and background climate conditions at a fraction of the computational cost (e.g., Meuer et al. 2024). By training these surrogates on existing super-ensembles, such as E19C or EVA40, future studies could extend the available simulations to more comprehensively sample the forcing–response space, improving our ability to identify statistically rare but physically plausible regional responses residing in the tails of internal climate variability.

More broadly, AI-based methods offer opportunities that extend well beyond emulation. Super-resolution techniques provide a complementary pathway for generating high-resolution climate fields from coarse-resolution simulations, while data mining and pattern-recognition algorithms can extract subtle signals from the rapidly growing volumes of climate-model output. Coupled with emerging approaches in explainable AI and causal inference, these methods offer new opportunities to identify coherent dynamical structures, constrain physically meaningful response pathways, and ultimately improve the interpretation of volcanic climate impacts.

### Integrated proxy-model frameworks

We must move toward a synthesis where models and data are no longer treated as independent entities, but as complementary constraints within a unified framework.

Instead of treating reconstructions as perfect validation targets for free-running models, Paleoclimate Data Assimilation (PDA) allows us to ingest proxy data and early instrumental observations directly into simulations. This effectively constrains the simulated climate toward a dynamically consistent historical state (e.g., the phase of the AMOC or NAO), isolating the volcanic signal within a physically consistent background. Significant advancements in this area are exemplified by the EKF400 and ModE-RA paleo-reanalyses (Franke et al. 2017; Valler et al. 2020). These products demonstrate that blending atmospheric model ensembles with documentary data and early instrumental records can successfully reproduce regional climate mechanisms—such as the weakened West African Monsoon or specific European temperature anomalies—that free-running models often miss (Brönnimann et al. 2019a, b; Valler et al. 2024).

Looking ahead, future proxy-model comparative assessments must fully account for the uncertainties inherent in climate-proxy records. We must move from a truth-centered paradigm toward ensemble consistency (Bothe et al. 2013a, b). Rather than validating against a fixed target, we must determine whether models and data provide a consistent representation of the unknown true climate evolution, i.e., evaluate the likelihood that a reconstructed anomaly resides within an ensemble phase space. This requires explicitly treating climate reconstructions as uncertain estimates rather than deterministic targets, ensuring that the interpretation of events such as 1816 remains robust to potential proxy biases and chronological uncertainties.

Integrated proxy–model frameworks therefore provide a natural pathway from deterministic model validation toward probabilistic attribution.

### Shifting paradigms: from the past to the future

Our observational and conceptual benchmarks must evolve to bridge the gap between paleoclimate and future risks.

The Hunga Tonga-Hunga Ha’apai eruption of 2022 demonstrated that volcanic forcing can operate through new radiative and chemical pathways, such as an exceptionally large water vapor injection (APARC 2025). We must expand our conceptual framework beyond the sulfate-only Pinatubo template.

The Hunga Tonga eruption also invites us to revisit long-standing assumptions about unidentified past eruptions. For instance, the 1809 unidentified eruption remains a “climatic cold case” (Timmreck et al. 2021) and has traditionally been modeled as a standard stratospheric sulfate event. While existing evidence, including recent explainable-AI analyses, points toward a northern extratropical fingerprint (Meuer et al. 2024), the Hunga Tonga case illustrates that non-standard eruption pathways—such as water-vapor rich submarine eruptions, halogen emissions (Aquila et al. 2013; Brenna et al. 2020; Staunton-Sykes et al. 2021), or ash-mediated aerosol evolution (Zhu et al. 2020)—may need to be considered when interpreting poorly constrained events.

Furthermore, we must apply the principles of near-term climate predictability to the study of individual volcanic events. In this framework, we shift from a deterministic view to a probabilistic one: as we have seen, for a specific eruption, the ensemble mean is often a poor predictor of regional impacts, as it masks the dominant role of internal variability and initial condition dependencies in shaping the spatial distribution of anomalies. Therefore, rather than deterministic reconstructions of past events, our objective should shift toward quantifying the conditional probability of a specific spatial pattern emerging from a given volcanic forcing, considering the internal state of the climate system at the time of the eruption.

Our data and recent event attribution studies (Schurer et al. 2019) suggest that while a Tambora-scale forcing makes extreme regional cooling significantly more probable, the specific sub-regional expression—such as the intense Central European anomaly of 1816—is a highly variable outcome. This implies that other Tambora-scale eruptions in the past may have produced equally severe cooling, but centered over different regions depending on the atmospheric circulation state at the time. Recognizing that volcanic forcing shifts the odds of regional crises without strictly dictating their location is essential for a more robust assessment of volcanic risk and for a physically consistent interpretation of the paleoclimate record.

While volcanic eruptions remain inherently unpredictable, our capacity to anticipate the impacts of a specific future event—such as a Tambora-scale eruption—is further complicated by the fact that the mean state of the climate system is itself variable and uncertain. A future eruption will unfold in an atmosphere with altered physical properties, which will fundamentally change the constraints on the resulting volcanic forcing.

For example, anthropogenic climate change is modifying the tropospheric lapse rate and increasing the tropopause height, factors that directly modulate volcanic plume dynamics (Aubry et al. 2021). This implies that a sulfur injection of the same magnitude and intensity as Tambora could result in a significantly different stratospheric aerosol burden depending on the background climate state, as the “threshold” for volcanic gases to penetrate the stratosphere shifts. Consequently, future volcanic risk depends not only on unknown initial conditions (such as the phase of ENSO, the NAO, or AMOC), but also on changes in the climate system’s sensitivity to volcanic forcing. In this evolving system, the same volcanic input may yield a substantially different climatic response, requiring us to move beyond historical analogues to fully grasp the spectrum of future volcanic hazards (Chim et al. 2023).

This principle applies equally to the deep past, where eruptions occurred under mean climatic states significantly different from those of the historical period. For instance, the volcanic forcing-response mechanism during glacial maxima or greenhouse climates was likely modulated by distinct background conditions in atmospheric stability and ocean circulation. Recognizing these dependencies suggests that even well-constrained eruptions from the paleoclimate record represent individual realizations of a dynamic interplay between volcanic forcing and an ever-evolving Earth system.

### A call to the Geoscience community

Deciphering the volcano–climate puzzle requires a fundamental shift from isolated disciplinary efforts toward a coordinated, interdisciplinary framework. Although volcanism has traditionally been treated as an external forcing of climate, the relationship is increasingly recognized as bidirectional. Climate change directly affects volcanic systems; for instance, the melting of ice sheets like the Vatnajökull in Iceland can lead to isostatic uplift, potentially altering magmatic pressures and triggering changes in regional volcanic activity (Compton et al. 2015; Aubry et al. 2022). The lessons of the early-nineteenth-century volcanic cluster highlight the need for a holistic approach to Earth system research, integrating atmospheric physics, volcanology, solid-Earth processes and magmatic dynamics within a unified Earth system perspective.

Filling in the next pieces of this puzzle will require the collective expertise of volcanologists, climate modelers, paleoclimatologists, geophysicists, and historians. The framework outlined here provides a basis for the geoscience community to address the multifaceted challenges posed by volcanic forcing in a changing climate. Only through such coordinated efforts can we refine our understanding of past eruptions, improve our ability to anticipate future volcanic impacts, and ultimately achieve the full integration of volcanic forcing within Earth system science.

## Data Availability

All data analysed in this study were obtained from previously published sources and are publicly available, including through the Earth System Grid Federation (ESGF). Post-processed data used to generate the figures are available from the corresponding author upon reasonable request.

## Declarations

### Collection

This manuscript has been submitted to the collection “Geosciences: next generation Earth telescope” of “Rendiconti Lincei. Scienze Fisiche e Naturali”.
